# Factors associated with the interruption of exclusive breastfeeding and weaning in premature infants after hospital discharge: a prospective cohort study^1^

**DOI:** 10.1590/1518-8345.7740.4831

**Published:** 2026-03-30

**Authors:** Mariana Lamante Bueno, Júlia Carneiro Godoy de Sousa, Vinícius de Sousa Monteiro, Alinne Almeida Sousa de Sá, Ana Karina Marques Salge Mendonça, Karina Machado Siqueira, Rafael Alves Guimarães, Edilaine Giovanini Rossetto, Thaíla Corrêa Castral

**Affiliations:** 1 Universidade Federal de Goiás, Faculdade de Enfermagem, Goiânia, GO, Brazil.; 2 Scholarship holder at the Coordenação de Aperfeiçoamento de Pessoal de Nível Superior (CAPES), Brazil.; 3 Secretaria Municipal de Saúde, Vigilância Epidemiológica de Óbitos Fetais e Infantis, Goiânia, GO, Brazil.; 4 Universidade Estadual de Londrina, Curso de Enfermagem, Londrina, PR, Brazil.

**Keywords:** Breast Feeding, Weaning, Premature Infant, Patient Discharge, Risk Factors, Neonatal Nursing.

## Abstract

**Objective:**

to estimate the incidence of exclusive breastfeeding and weaning in premature infants and identify risk factors after hospital discharge.

**Method:**

prospective cohort study with 237 participants. Factors associated with breastfeeding discontinuation and weaning were analyzed using Poisson regression.

**Results:**

after discharge, 35.3% (95% CI: 29.6-41.8) interrupted breastfeeding and 9.8% (95% CI: 6.5-14.2) weaned. The following factors were associated with breastfeeding cessation: maternal age (RR=0.70; 95% CI: 0.49-0.99); education (elementary school: RR=1.68; 95% CI: 1.00-2.80; high school: RR=1.79; 95% CI: 1.20-2.66); breast stimulation (>6 h: RR=1.88; 95% CI: 1.13-3.13; no stimulation: RR=1.69; 95% CI: 0.98-2.88); hospitalization (RR=1.85; 95% CI: 1.10-3.08); first feeding (>24 h: RR=0.61; 95% CI: 0.38-0.99; did not breastfeed: RR=0.66; 95% CI: 0.43-1.01); and breastfeeding at discharge (RR=3.64; 95% CI: 2.45-5.40). Weaning was associated with not breastfeeding at discharge (RR=3.64; 95% CI: 2.45-5.40).

**Conclusion:**

maternal age, education, hospitalization, and breastfeeding at discharge influenced the interruption of breastfeeding. The absence of breastfeeding at discharge increased the risk of weaning. Early interventions, family support, and policies are essential for maintaining breastfeeding.

## Highlights

**(1)** Monitoring the breastfeeding rate in premature infants is crucial. **(2)** The incidence of breastfeeding decreased after hospital discharge. **(3)** Understanding the risks of breastfeeding cessation helps public policy.

## Introduction

Prematurity is the leading cause of neonatal mortality^1^. International data reveal the birth rate of 13.4 million premature infants (PT, in Portuguese) worldwide, with prematurity rates ranging from 4 to 16% among countries. Approximately 85% of premature (PT) births occur between 32 and 37 weeks of gestation and generally do not require intensive care^2^.

In 2021, Brazil recorded 2,677,101 live births, of which 11.3% were preterm, with the majority (85.9%) having a gestational age of 32-36 weeks. In the Midwest, 229,296 live births were recorded, of which 11.4% were preterm, with the majority (86.1%) also in the same age range^3^.

Premature birth has multiple causes (gestational, maternal, socioeconomic, and environmental)^4^ ^-^ ^6^. By 2030, one of the Sustainable Development Goals is to eradicate preventable neonatal deaths and reduce newborn deaths and stillbirths^7^.

Small or sick PTs are at greater risk of developing infectious diseases, developmental and growth delays, and mortality in early childhood. Exclusive breastfeeding (EBF) is an effective intervention for preventing these outcomes^8^, as human milk is essential for protecting against infectious and chronic diseases, reducing the risk of neurodevelopmental disorders, and promoting mother-child bonding^9^ ^-^ ^11^.

Given the importance of breastfeeding and human milk, public policies and strategies to promote EBF have been developed. The Baby-Friendly Hospital Initiative (BFHI) is one of the main international strategies for promoting and protecting EBF^12^. However, the BFHI was developed for full-term newborns and does not target specific strategies for preterm infants. In 2020, the World Health Organization (WHO) updated the document, including additional guidelines for small, sick, and/or preterm newborns, emphasizing the mother’s constant presence and breastfeeding (BF)^7^.

Brazilian studies indicate that the incidence of exclusive breastfeeding in preterm infants is 81.4% at hospital discharge, declining to 75% at 15 days^12^ and 66.4% between 7 and 15 days after discharge^13^. This progressive reduction reflects the challenges inherent in breastfeeding PTs, associated with factors such as physiological immaturity^14^, restrictive hospital practices, and family dysfunction^13^.

However, this scenario was aggravated by the COVID-19 pandemic, which altered maternal and neonatal care practices, compromising BF indicators. International^15^ and national evidence has documented restrictions such as the prohibition of skin-to-skin contact and the exclusion of companions^16^, factors that, combined with inadequate breastfeeding support and the absence of rooming-in, have reduced EBF rates^15^.

Although these impacts are recognized, there is a critical knowledge gap regarding EBF in PTs in the post-pandemic scenario, particularly after hospital discharge. Existing Brazilian studies predate the pandemic or are geographically limited.

Knowing the rates of EBF and the factors that facilitate or hinder its maintenance is crucial to improving nursing care in neonatal units and its articulation with primary care. Investigating these indicators and the causes of EBF interruptions is essential for planning post-pandemic public policies that strengthen breastfeeding support, enabling monitoring and the timely response to identified needs.

The objective of this study is to estimate the incidence of exclusive breastfeeding and weaning in premature infants and to identify risk factors after hospital discharge.

## Method

### Study design

This is a prospective cohort study. The study report followed the recommendations of the Strengthening the Reporting of Observational Studies in Epidemiology (STROBE) checklist^17^. The incidence of EBF at hospital discharge and up to 15-30 days after hospital discharge was examined among preterm infants exposed and not exposed to risk factors (maternal and neonatal variables).

### Context

The study was carried out in maternity hospitals in Londrina-PR, with 24 neonatal beds and 17 rooming-in beds; and in a maternity hospital in Goiânia-GO, with 25 neonatal beds and 63 beds in rooming-in. The two maternity hospitals are a reference in the care of high-risk pregnancies and in the Kangaroo Method (KM), offering all stages of the progressive line of neonatal care. Both hold the Baby-Friendly Hospital designation and provide care exclusively through the Unified Health System. Data collection took place between March 2022 and February 2023.

### Population

The study population consisted of mothers and PTs (<37 weeks of gestational age) admitted to the joint accommodation and neonatal units of the two centers.

### Eligibility criteria

The inclusion criteria were postpartum women with PT infants hospitalized in rooming-in, neonatal intensive care, or neonatal intermediate care units who had a smartphone with internet access, could read and understand Portuguese, and wanted to breastfeed. Exclusion criteria were postpartum women with contraindications for breastfeeding, a diagnosis of COVID-19, or newborns with clinical conditions that prevented them from receiving human milk. Mothers whose newborns did not have feeding data after hospital discharge were excluded.

### Sampling and sample

A non-probabilistic convenience sampling method was used, a technique recommended in cohort studies when the population is difficult to access^18^, such as premature newborns after discharge. The sample calculation was performed for an analytical cohort study^19^ ^-^ ^20^, using the following parameters: type I error probability or significance level of 5% (α=0.05), probability of type II error or statistical power of 80% (1-β=0.2), loss rate of 20.0%, and a minimum detectable Relative Risk (RR) of 1.5 to represent a clinically significant difference for the main exposure variables investigated. A cumulative incidence of EBF of 53.8% in the unexposed group of the main independent variables was also adopted as a parameter, a value obtained in a previous study with a similar population, in which 93.8% (15/16) of the independent variables had an incidence equal to or greater than 53.8% in unexposed groups^13^. Finally, Fleiss’s continuity correction was applied in the sample calculation^20^, resulting in an estimated minimum sample of 136 newborns for evaluation between 15 and 30 days after hospital discharge.

### Study variables

Two outcome variables were selected: interruption of EBF and weaning 15-30 days after hospital discharge. Weaning was considered the interruption of BF in the week before contact with the mother or recording in the medical record.

The exposure variables were distributed into blocks according to their proximity to the outcomes, in accordance with an adapted theoretical approach^13^ ^,^ ^21^. The first theoretical block (distal) included the variables maternal age and maternal education, while the second block (intermediate) included the variable previous breastfeeding experience. The third block (proximal) included the type of delivery (cesarean section), birth weight, gestational age, EBF at discharge, time of first breast stimulation, age at first breastfeeding, and length of hospitalization.

Censoring was considered when: 1) the mother did not respond to messages or calls; 2) the mother stated that she did not wish to continue in the study; 3) the infant died or was transferred to another hospital. The mother and infant were excluded from the study only when the mother withdrew her consent. 

### Instruments used in data collection

Part of the data collection was carried out using OpenDataKit (ODK). This platform, after discharge, allows offline data collection on smartphones or tablets and sends data to a server when an internet connection is available. Registration forms for participants and questions about the NB’s feeding (milk type and feeding method) at hospital discharge and after discharge were developed by researchers on this platform and underwent a pilot test. In the “*AmamentaCoach*” app, developed by researchers from the School of Nursing at the State University of Londrina, forms about the NB’s feeding were also included to facilitate data collection. The data was exported to Power BI.

A research agenda was developed in an Excel spreadsheet to track participants. Data from the ODK and Power BI platforms were exported and integrated into an Excel spreadsheet, forming a unified database.

### Data collection procedure

Data collection occurred at three moments: birth, hospital discharge, and after hospital discharge. The eligible mother was approached between 72 and 120 hours after giving birth. After giving their consent, they were invited to install the “*AmamentaCoach*” app on their mobile device.

Upon discharge from the hospital and within 15 to 30 days thereafter, the mother received notifications via the app to inform her of her discharge and to answer questions about infant feeding. Those who did not respond via the app received messages via WhatsApp (three attempts) and phone calls (three attempts). When contact was not possible, the information was sought in the maternity ward’s electronic medical records.

### Ethical aspects

All ethical aspects provided for in Resolution No. 466/2012 were ensured. The study was approved by the Research Ethics Committee (No. 27703419.8.0000.5231).

Eligible mothers were invited to participate in the study, and after being informed of its objectives and giving their consent, the Free and Informed Consent Form was read together and signed. For adolescent mothers, the Assent Form and the Free and Informed Consent Form were provided to the guardian.

### Data processing and analysis

The data were imported from Excel and analyzed using Stata software, version 17.0. The normality of the quantitative variables was tested using the Kolmogorov-Smirnov test with Lilliefors’ correction. In the descriptive analysis, quantitative variables were reported as mean and standard deviation (SD), along with median, 25th percentile (P25), and 75th percentile (P75) due to non-normality. Qualitative variables were presented as absolute (n) and relative (%) frequencies. A descriptive analysis of the cumulative incidence of outcomes (discontinuation of EBF and weaning after discharge) was also performed.

Next, bivariate analysis was performed using Poisson regression with robust variance to assess the magnitude of the association between each exposure variable and the outcomes. This strategy was used to select the variables for the multiple regression model. Variables with a p-value <0.25 in this analysis were included in various regression models^22^ ^-^ ^23^. The results of the bivariate analysis were presented as Unadjusted Relative Risk (RR) and the respective 95% confidence interval (95% CI). The results of the multiple regression model were presented as Adjusted Relative Risk (ARR) and the respective 95% CI, considering p<0.05.

## Results

Of the 291 initial participants, five newborns died, one newborn was transferred to another hospital, one newborn was not discharged from the hospital, one mother was unable to download the data collection app, and two mothers could not be contacted at the time of discharge from the NICU. After hospital discharge, there was no contact with the mothers of 42 newborns, and two mothers withdrew from the study, totaling 237 mother-child pairs with complete feeding data 15-30 days after hospital discharge as the final sample. Table 1 shows the sociodemographic and clinical characteristics of the participants. Most mothers were between 20 and 34 years old (67.1%); 67.1% had completed high school, and 82.7% lived with their partner. Among pregnant women, 40.5% were primiparous, 41.4% planned their pregnancy, 76.4% had six or more prenatal consultations, 22.4% received information about breastfeeding (BF), and 53.2% had previous experience with BF. Regarding delivery, 59.9% had complications, 38.8% had vaginal delivery, 40.9% had skin-to-skin contact up to 72 hours after delivery, and 41.8% breastfed within the first 24 hours.

**Table 1:** Characteristics of mother-child pairs participating in the study cohort (n = 237). Goiânia, GO; Londrina, PR, Brazil, 2022-2023

Variables	n* (%)^†^
Maternal age (years)	
<19	28 (11.8)
20-34	159 (67.1)
>35	50(21.1)
Mother’s education	
Incomplete or complete elementary education	35 (14.8)
Incomplete or complete high school education	159 (67.1)
Higher education	43 (18.1)
Lives with partner	196 (82.7)
Planned pregnancy	98 (41.4)
Primiparous	96 (40.5)
Had six or more prenatal consultations	181 (76.4)
Had complications during pregnancy	142 (59.9)
Received information about breastfeeding during prenatal care	53 (22.4)
Has previous experience with breastfeeding	126 (53.2)
Vaginal delivery	92 (38.8)
Skin-to-skin contact up to 72 hours after delivery	97 (40.9)
First breast stimulation	
<6 hours after delivery	85 (35.9)
>6 hours after delivery	121 (51.1)
No stimulation until the fifth day after delivery	31 (13.1)
Birth weight (grams)	
> 2,500	85 (35.9)
2,500-1,500	122 (51.5)
< 1,500	30 (12.7)
The period in which the newborn breastfed for the first time	
< 24 hours after birth	99 (41.8)
> 24 hours after birth	51 (21.5)
Did not breastfeed until the fifth day of life	87 (36.7)
Exclusive breastfeeding at hospital discharge	170 (71.7)
Gestational age at birth (weeks)	
Mean (Standard deviation)	34.19 (2.2)
Median (25th-75th percentile)	35 (33.5-36)
Total length of hospital stay (days)	
Mean (Standard deviation)	14.30 (18.6)
Median (25th-75th percentile)	6 (4-16.5)

*n = Number; ^†^% = Frequency

**Figure 1:**
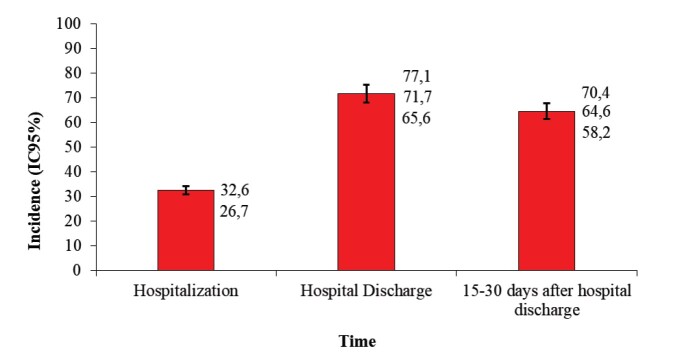
Indicators of exclusive breastfeeding in the first five days of life, at hospital discharge, and 15-30 days after hospital discharge of preterm infants (n = 237). Goiânia, GO; Londrina, PR, Brazil, 2022-2023

The newborns had a mean gestational age of 34.19 weeks, and 51.5% weighed between 1,500 and 2,500 g. Most mothers began breast stimulation within 6 hours of delivery (51.1%). The mean hospital stay for newborns was 14.3 days. In the first five days, 32.6% of preterm infants were on EBF, increasing to 71.7% at discharge and decreasing to 64.6% after 15-30 days (Figure 1). The cumulative incidence of early weaning was 9.8% (95% CI: 6.5-14.2%).

In the bivariate analysis, maternal and neonatal variables, including maternal age, education, breastfeeding experience, type of delivery, birth weight, and EBF at discharge, were significantly associated with EBF discontinuation. For BF weaning, maternal and neonatal variables, including breastfeeding experience, cesarean delivery, skin-to-skin contact, birth weight, and EBF at discharge, were significant (Table 2). 

**Table 2:** Incidence rate of interruption of exclusive breastfeeding and weaning, and crude relative risk (RRg) in preterm infants 15-30 days after hospital discharge, according to exposure variables (n = 237). Goiânia, GO; Londrina, PR, Brazil, 2022-2023

Maternal variables	Interruption of EBF*		RRg^†^(CI95%^‡^)	p-value^§||^	Weaning		RRg^†^(CI95%^‡^)	p-value^§||^
	No (n=153)	Yes (n=84)			No (n=214)	Yes (n=23)		
Age (years)								
<19	7 (25.0)	21 (75.0)	0.63 (0.32-1.23)	0.179	25 (89.3)	3 (10.7)	1.14 (0.35-3.68)	0.832
20-34	63 (39.6)	96 (60.4)	1.00		144 (90.6)	15 (9.4)	1.00	
>35	14 (28.0)	36 (72.0)	0.70 (0.44-1.15)	0.161	45 (90.0)	5 (10.0)	1.06 (0.40-2.78)	0.906
Education								
Up to elementary school	14 (40.0)	21 (60.0)	1.56 (0.81-3.00)	0.180	145 (88.7)	18 (11.3)	0.82 (0.14-4.64)	0.822
Up to high school	59 (37.1)	100 (62.9)	1.45 (0.84-2.51)	0.185	40 (93.0%)	3 (7.0)	1.62 (0.50-5.3)	0.420
Higher education or above	11 (25.6)	32 (74.4)	1.00		40 (93.0)	3 (7.0)	1.00	
Lives with partner								
No	13 (31.7)	28 (68.3)	0.88(0.54-1.42)	0.592	36 (87.8)	5 (12.2)	1.33 (0.52-3.38)	0.552
Yes	71 (36.2)	125 (63.8)	1.14 (0.70-1.85)	0.592	178 (9.8)	18 (9.2)	1.00	
Had > 6 prenatal consultations								
No	21 (37.5)	35 (62.5)	1.07 (0.73-1.60)	0.710	50 (89.3)	6 (10.7)	1.14 (0.47-2.76)	0.770
Yes	63 (34.8)	118 (65.2)	1.00		164 (90.6)	17 (9.4)	1.00	
Received information about breastfeeding during prenatal care								
No	67 (36.4)	117 (63.6)	1.13 (0.73-1.76)	0.569	164 (89.1)	20 (10.9)	1.92 (0.59-6.22)	0.277
Yes	17 (32.1)	36 (67.9)	1.00		50 (90.3)	3 (5.7)	1.00	
Previous experience with breastfeeding								
No	44 (39.6)	67 (60.4)	1.25 (0.88-1.76)	0.207	96 (86.5)	15 (13.5)	2.12 (0.93-4.83)	0.071
Yes	40 (31.7)	86 (68.3)	1.00		118 (93.7)	8 (6.3)	1.00	
Cesarean delivery								
No	65 (44.8)	80 (55.2)	2.17 (1.40-3.37)	0.001	126 (86.9)	19 (13.1)	3.01 (1.05-8.60)	0.039
Yes	19 (20.7)	73 (79.3)	1.00		88 (95.7)	4 (4.3)	1.00	
Skin-to-skin contact up to 72 hours after delivery								
No	52 (37.1)	88 (62.9)	1.12 (0.79-1.61)	0.515	122 (87.1)	18 (12.9)	2.49 (0.95-6.50)	0.062
Yes	32 (33)	65 (67)	1.00		92 (94.8)	5 (5.2)	1.00	
First breast stimulation (hours)								
<6 hours after delivery	15 (17.6)	70 (82.4)	1.00		83 (97.6)	2 (2.4)	1.00	
>6 hours after delivery	52 (43.0)	69 (57.0)	2.43 (1.47-4.03)	0.001	109 (90.1)	12 (9.9)	4.21 (0.96-18.40)	0.056
did not stimulate until 5 days after delivery	17 (54.8)	14 (45.2)	3.10 (1.77-5.44)	<0.001	22 (71.0)	9 (29.0)	12.3 (2.81-54.15)	0.001
**Neonatal Variables**	Interruption of EBF*		**RRg^†^(CI95%^‡^)**	**p-value^§||^**	**Weaning**		**RRg^†^(CI95%^‡^)**	**p-value^§||^**
	**No (n=153)**	**Yes (n=84)**			**No (n=214)**	**Yes (n=23)**		
Birth weight (grams)								
> 2,500	19 (22.4)	66 (77.6)	1.00		81 (95.3)	4 (4.7)	0.71 (0.22-2.31)	0.578
2,500-1,500	43 (35.2)	79 (64.8)	1.58 (0.99-2.50)	0.055	114 (93.4)	8 (6.6)	1.00	
< 1,500	22 (73.3)	8 (26.7)	3.28 (2.08-5.15)	<0.001	19 (63.3)	11 (36.7)	5.59 (2.46-12.70)	<0.001
Gestational age at birth (weeks)								
<28	1 (20)	4 (80)	0.61 (0.10-3.58)	0.585	4 (80)	1 (20)	3.06 (0.49-19.03)	0.231
28-32	13 (72.2)	5 (27.8)	2.20 (1.56-3.11)	<0.001	10 (55.6)	8 (44.4)	6.79 (3.29-14.02)	<0.001
32-37	70 (32.7)	144 (67.3)	1.00		200 (93.5)	14 (6.5)	1.00	
Length of hospital stay (days)								
< 7	28 (23.3)	92 (76.7)	1.00		117 (97.5)	3 (2.5)	1.00	
7-14	13 (24.5)	40 (75.5)	1.05 (0.59-1.87)	0.865	50 (94.3)	3 (5.7)	2.26 (0.47-10.9)	0.308
> 14	43 (67.2)	21 (32.8)	2.88 (1.99-4.16)	<0.001	47 (73.4)	17 (26.6)	10.62 (3.22-35.0)	<0.001
Age at which newborn first breastfed								
< 24 hours after delivery	28 (28.3)	71 (71.7)	1.00		96 (97)	3 (3)	1.00	
> 24 hours after delivery	16 (31.4)	35 (68.6)	1.11 (0.66-1.85)	0.693	46 (90.2)	5 (9.8)	3.23 (0.80-13.03)	0.099
did not breastfeed until the fifth day of life	40 (46.0)	47 (54.0)	1.62 (1.10-2.40)	0.014	72 (82.8)	15 (17.2)	5.68 (1.70-19.04)	0.005
It was in EBF* when I was discharged from the hospital.								
No	55 (82.1)	12 (17.9)	4.81 (3.39-6.83)	<0.001	48 (71.6)	19 (28.4)	12.05 (4.24-34.19)	<0.001
Yes			1.00		166 (97.6)	4 (2.4)	1.00	

*EBF = Exclusive breastfeeding; ^†^RRg = Unadjusted relative risk; ^‡^ CI95% = 95% confidence interval; ^§^p=0.05; ^||^Wald chi-square test

In the hierarchical multiple analysis (Table 3), maternal age > 35 years reduced the risk of EBF discontinuation by 0.30 times. Low educational attainment increased the risk: 1.68 times for elementary school education and 1.79 times for high school education. Breast stimulation after 6 hours or absence of stimulation increased the risk by 1.88 and 1.69 times, respectively. Length of hospitalization increased the risk by 1.85 times. The first feeding after 6 hours increased the risk by 1.61 times, and the absence of feeding until the fifth day increased the risk by 1.66 times. Not being on EBF at discharge increased the risk by 3.64 times. 

In the hierarchical multivariate analysis, not being in AME at hospital discharge increased the risk of weaning from BF 15-30 days after discharge by 6.13 times, as demonstrated in Table 4.

**Table 3:** Multivariate analysis using Poisson regression with a hierarchical model for discontinuation of exclusive breastfeeding 15-30 days after hospital discharge of preterm infants and associated factors (n = 237). Goiânia, GO; Londrina, PR, Brazil, 2022-2023

Variables	Model I		Model II		Model III	
	aRR*	CI95%^†^	aRR*	CI95%^†^	aRR*	CI95%^†^
Block 1						
Mother’s age (years)						
< 19	0.57 (0.28-1.14)		0.50 (0.25-1.02)		0.63 (0.36-1.12)	
20-34	1.00		1.00		1.00	
> 35	0.71 (0.43-1.16)		0.75 (0.45-1.25)		0.70 (0.50-1.0)^‡^	
Mother’s education						
Up to elementary school	1.75 (0.90-3.40)		1.95 (1.0-3.7)^||^		1.68 (1.00-2.80)^‡^	
Up to high school	1.50 (0.85-2.62)		1.6 (0.9-2.7)		1.79 (1.20-2.66)^‡^	
Higher education or above	1.00		1.00		1.00	
**Block 2**						
Previous experience with breastfeeding						
No			1.38 (1.0-1.9)		1.26 (0.94-1.69)	
Yes			1.00		1.00	
**Block 3**						
Cesarean delivery						
No					1.00	
Yes					1.26 (0.85-1.88)	
First breast stimulation						
< 6 h postpartum					1.00	
>6 h postpartum					1.88 (1.13-3.13)^‡^	
Did not stimulate until the fifth day after delivery					1.69 (0.98-2.88)^‡^	
Birth weight (grams)						
> 2,500					1.00	
2,500-1,500					1.24 (0.72-2.14)	
< 1,500					0.89 (0.61-1.28)	
Gestational age at birth (weeks)						
< 28					0.37 (0.10-1.32)	
28-32					0.99 (0.71-1.37)	
32-37					1.00	
Length of hospital stay						
< 7 days					1.00	
7-14 days					0.97 (0.58-1.63)	
> 14 days					1.85 (1.10-3.08)^‡^	
Age at which he/she first breastfed						
< 24 hours after delivery					1.00	
> 24 hours after delivery					0.61 (0.38-0.99)^‡^	
did not breastfeed until the fifth day of life					0.66 (0.43-1.01)^‡|^	
Exclusive breastfeeding at hospital discharge						
No					3.64 (2.45-5.40)^§^	
Yes					1.00	

*aRR = Adjusted relative risk; ^†^CI95% = 95% Confidence interval; ^‡^p<0.05 Wald chi-square test; ^§^p<0.001

**Table 4:** Multivariate analysis using Poisson regression with a hierarchical model for weaning from breastfeeding 15-30 days after hospital discharge of preterm infants and associated factors (n = 237). Goiânia, GO; Londrina, PR, Brazil, 2022-2023

Variables	Distal		Intermediary		Proximal	
	ARR*	CI95%^†^	ARR*	CI95%^†^	ARR*	CI95%^†^
**Block 1**						
Previous experience with breastfeeding						
No			2.13 (0.93-4.84)		1.97 (0.96-4.02)	
Yes			1.00		1.00	
**Block 2**						
Cesarean delivery						
No					1.00	
Yes					1.23 (0.42-3.56)	
First breast stimulation						
<6 hours after delivery					1.00	
>6 hours after delivery					1.75 (0.44-6.89)	
No stimulation until the fifth day after delivery					2.47 (0.62-9.83)	
Birth weight (grams)						
> 2,500					1.00	
< 1,500					1.50 (0.55-4.07)	
> 2,500					2.72 (0.64-11.4)	
Gestational age at birth (weeks)						
< 28					0.85 (0.19-3.77)	
28-32					1.84 (0.68-4.96)	
32-37					1.00	
Total length of hospital stay (days)						
< 7					1.00	
7-14					1.85 (0.36-9.35)	
> 14					2.98 (0.64-13.72)	
Skin-to-skin contact up to 72 hours after delivery.						
No					1.50 (0.51-4.35)	
Yes					1.00	
Exclusive breastfeeding at hospital discharge						
No					6.13 (2.43-15.40)^‡^	
Yes					1.00	

*aRRa = Adjusted relative risk, ^†^CI95% = Confidence interval, ^‡^p<0.001 Wald chi-square test

## Discussion

The benefits of BF are numerous, both for the mother/family, and the infant^8^ ^-^ ^10^. For the PT group, the benefits extend and can directly impact the clinical response of this newborn admitted to a neonatal unit, reducing infections, among other complications, in addition to human milk being essential for PT development. There are many difficulties in keeping this infant on BF, and they extend beyond hospital discharge^24^.

In this study, a reduction in the incidence of EBF was observed after hospital discharge (from 71.7% to 64.2%). Studies in other cities and countries show differences in EBF indicators at discharge and after hospital discharge, highlighting the importance of understanding the context and risk factors when comparing these data.

A study in Goiânia, GO, with 113 PTs, also showed a decrease in the incidence of EBF 7-15 days after discharge (from 81.4% to 66.4%)^13^. In Recife, Pernambuco, a cross-sectional study in two public hospitals with 108 PTs found an incidence of EBF of 85.2% at discharge, 75% at 15 days, and 46.3% at 30 days^25^. A cohort study in Rio de Janeiro, RJ, followed 1,003 infants (22.5% were PT). The prevalence of EBF at hospital discharge was only 14.3% among PT^24^.

After hospital discharge, the EBF index was lower than that found in the cross-sectional study conducted in Recife with 108 PTs^25^ and in the prospective cohort study conducted in Goiânia with 113 PTs^13^ 15-30 days after discharge; however, it was higher than in the Recife study^25^ 30 days after hospital discharge. All analyses were conducted in Baby-Friendly hospitals, and data collection in our study took place during the COVID-19 pandemic.

In Greece, a retrospective study of 279 mothers and newborns, of whom 122 (43.7%) were preterm (15.4% with a gestational age < 32 weeks and 28.3% late preterm), showed a prevalence of EBF of 58.1% in the first month. The average duration of EBF was 2 months in PT and 3 months in term infants (p=0.002). Preterm infants had a 1.64% higher risk of discontinuing EBF^26^. In Shanghai (China), a cohort study of 500 PT showed that 19% were breastfed one month after discharge. During hospitalization in the neonatal unit, mothers were unable to breastfeed directly, and human milk was expressed and administered by professionals^27^.

In Australia, a cohort study of 270 late PTs showed that 74% of babies were discharged on EBF, reducing to 41% at 6 weeks of corrected age^28^. In Sweden, a study of 29,445 PTs (6.6% extreme, 16% very premature, and 18% moderate) found an EBF incidence of 59% in 2004 and 45% in 2013^29^.

It was observed that the incidence of EBF at discharge^28^ ^-^ ^29^ ^)^ and after discharge^26^ ^-^ ^28^ ^)^ was higher in our study than in other countries. Despite the differences, EBF rates decrease significantly after hospital discharge, pointing to the challenges of maintaining EBF in this population, even in countries that have policies to encourage breastfeeding, such as Brazil and Sweden.

These numbers, although significant, are still below the WHO recommendations of 90% at discharge and 70% of EBF in the first 6 months^30^. However, there are no specifications for these targets for PTs, despite evidence of greater vulnerability in these babies to early weaning.

Global data revealed that between 2015 and 2021, only 48% of infants under six months of age were exclusively breastfed worldwide, and 16% of annual infant deaths occur due to inadequate breastfeeding^30^.

An initiative by the United Nations Children’s Fund (UNICEF) and WHO identified priorities for countries to protect and promote breastfeeding. Only 16% of countries have aligned their legislation with the International Code of Marketing of Breast-milk Substitutes, with Africa and Asia leading the way. Only 9% of countries meet the recommendation for paid maternity leave, with Oceania failing to meet it in 31% of cases. In 14% of countries, most births took place in Baby-Friendly Hospitals; 70% had community breastfeeding programs; 23% had emergency support; and 43% collected data on EBF over the five years^30^. In the World Breastfeeding Trends Initiative ranking, Brazil ranks 18th, behind Bangladesh (1^st^ place)^31^.

Improvement in these indicators is only possible if risk factors for weaning are identified and specific policies and actions are planned for each context. In this study, multivariate analysis revealed low maternal education as a risk factor and maternal age as a protective factor for discontinuation of EBF 15-30 days after hospital discharge of the PT infant. A study in Maceió, Alagoas, with 161 PTs on EBF at hospital discharge, found similar results, suggesting that women in this age group are more aware of the benefits of EBF, despite the associated obstetric risks^32^. Another study with 622 postpartum women in Porto Alegre, Rio Grande do Sul, found that maternal age ≥ 35 years was a protective factor for EBF. On the other hand, women between 26 and 34 years of age face challenges related to working life, such as the need to return to work, short maternity leave, and financial insecurity^33^.

A systematic review identified 36 factors associated with EBF interruption, with socioeconomic variables such as place of residence, education, and race considered distal factors. Maternal education was the most frequently investigated variable, with lower education often associated with EBF interruption^21^. In our study, low maternal education was also associated with a higher risk of EBF discontinuation 15-30 days after hospital discharge. However, we investigated only two distal variables (education and the mother’s age), which was a limitation of the study.

Low educational attainment can lead to difficulties in understanding guidelines and maintaining EBF^34^. In addition, mothers with more years of schooling are likely to have greater control in the workplace, receiving more support and encouragement for EBF for longer^35^.

Educational initiatives to improve understanding of breastfeeding are effective for initiating and maintaining EBF, particularly when delivered during pregnancy in primary health care centers or high-risk clinics. Two studies showed an increase in knowledge following educational initiatives (dialogue-based presentations, videos, posters), but did not analyze improvements in EBF rates^36^ ^-^ ^37^.

Multivariate analysis revealed associations with several proximal variables: time of first breast stimulation, age at first breastfeeding, length of hospitalization, and EBF at hospital discharge. The absence of EBF at discharge was the variable with the highest risk for discontinuation of EBF (3.64 times higher) and for weaning after discharge (6.13 times higher).

A cohort study involving 12 countries (Albania, Brazil, Bulgaria, Cyprus, Chile, Greece, Israel, Malta, Portugal, Spain, Turkey, and the United Kingdom) examined the impact of the pandemic on factors influencing breastfeeding rates, analyzing data from 5,612 women in high- and upper-middle-income countries. Risk factors for EBF included primiparity, infant age, prematurity, admission to the Neonatal Intensive Care Unit (NICU), lack of breastfeeding support during prenatal care and after discharge, and psychiatric treatment^38^.

Other studies have also found an association between discontinuation of EBF in PT and length of hospital stay^24^ ^,^ ^39^. In our study, most babies were hospitalized for less than 15 days. Longitudinal studies with more extended follow-up periods may shed more light on this relationship.

An effective, low-cost national policy to reduce hospitalization time is the Kangaroo Method (KM), a priority strategy for PT and low-weight babies. According to the Ministry of Health, one of the criteria for hospital discharge in the second stage of KM is EBF. KM reduces the time to separation between the newborn and parents, facilitates family bonding, supports clinical stabilization of the newborn, and ensures breastfeeding stimulation and maintenance. After discharge, these babies receive specialized follow-up by the team of a Basic Health Unit, in partnership with the maternity hospital of origin, during the first weeks at home^40^.

There is evidence that early breastfeeding and skin-to-skin contact in PT^26^ ^)^ and term^41^ contribute to maintaining EBF. In this study, less than half of PTs performed KM within 72 hours after delivery. It is noteworthy that only one participating NICU has an evidence-based protocol with criteria for initiating kangaroo care, which is generally subjective and linked to the baby’s clinical stability, potentially delaying its initiation.

Some studies have associated the feeding type of preterm infants in the NICU and the provision of human milk with the duration of EBF^26^ ^-^ ^27^ ^,^ ^41^, but we found no studies on the age at which preterm infants first breastfed or on the interruption of exclusive breastfeeding. Scientific evidence indicates that the baby›s stability should be the only criterion for initiating breastfeeding, as PTs can breastfeed early^42^. In our study, 87 (36.7%) of PTs did not breastfeed until the fifth day of life.

Previous experience with breastfeeding was the only intermediate factor in the hierarchical multivariate analysis. Half of the postpartum women (53.2%) had previous experience with breastfeeding. This variable showed an association with EBF interruption in the bivariate analysis but was not significant in the multivariate analysis, unlike other studies^26^ ^,^ ^41^.

A systematic review of 15 studies involving 16,579 mothers revealed that previous breastfeeding experience is associated with the initiation and duration of subsequent breastfeeding. The quality of this experience, if satisfactory and prolonged, positively influenced the duration of breastfeeding^43^. Future studies should investigate the quality and duration of previous breastfeeding experience as risk factors for EBF continuation.

Most mothers performed the first breast stimulation within 6 hours of delivery (51.1%) or did not do so until the interview (13.1%). A study of 263 PT mothers found a higher risk of EBF absence at hospital discharge when pumping began after 48 hours^39^. One study investigated the factors associated with late initiation of breast pumping (> 6 hours after delivery) in 129 mothers of preterm infants. Previous experience in the NICU, male sex of the baby, compromised maternal psychological well-being, cesarean section, and gestational age at birth were identified as risk factors for late pumping^44^.

It is recommended that appropriate guidance and care for postpartum women regarding milk expression be provided during pregnancy and in the first 3-6 hours after delivery, or as soon as possible^7^ ^,^ ^45^. Mothers should receive instructions and demonstrations on how to express milk manually or with a pump^42^. Human milk feeding significantly impacts early intestinal colonization in the PT, protecting against necrotizing enterocolitis^45^.

Planning and coordinating actions between hospital departments is essential to ensure early initiation of pumping and facilitate the efficient supply of human milk to PT. Regarding weaning from BF, 15-30 days after the PT’s hospital discharge, hierarchical multivariate analysis identified only one significant risk variable: not being on EBF at hospital discharge (6.13 times higher risk). Previous studies corroborate that the absence of EBF at discharge is associated with an increased risk of early weaning^25^ ^,^ ^46^. The provision of human milk in the first enteral feeding of the PT, when given as a supplement during hospitalization, was associated with weaning after hospital discharge in other studies, as postpartum women who were prescribed it during hospitalization questioned their ability to breastfeed^24^ ^,^ ^26^ ^-^ ^27^.

Several obstacles after hospital discharge can make it challenging to maintain EBF, such as adapting to the home routine, maternal perception of low milk supply, lack of family and professional support, early return to work, early introduction of complementary foods, and lack of information about the importance of EBF^24^ ^-^ ^25^ ^,^ ^41^. Future studies that follow these premature infants for an extended period after discharge are essential to investigate other factors that interfere with early weaning.

Healthcare professionals working in neonatal units should implement individualized interventions to promote PT development, protecting and encouraging breastfeeding from the outset, facilitating early skin-to-skin contact, and promoting its continuity^47^.

We understand that cultural differences and health policies influence breastfeeding outcomes for premature infants, integrating international evidence with local experiences, such as the Kangaroo Method in Brazil and variations in skin-to-skin contact practices. These disparities reveal a complex scenario of inequality in EBF rates. Given this the authors advocate the urgency of longitudinal and prospective nursing research to assess the impact of cultural and systemic factors and care practices on the duration of EBF after discharge. They also highlight the importance of investigating how prior breastfeeding experience and the care context affect maternal decisions, as well as of testing scientifically based, culturally adapted nursing interventions. 

The results of this study reinforce that implementing actions grounded in scientific evidence and existing policies can improve EBF indicators and reduce early weaning after hospital discharge of preterm infants, thereby favoring EBF until 6 months of age.

The crucial role of nurses in leading comprehensive care for PTs and their families, both in the NICU and after hospital discharge, is highlighted. The planning and implementation of actions to promote breastfeeding are fundamental responsibilities of these professionals^47^.

Assistance to PTs in hospitals with the Baby-Friendly title and the implementation of the MC probably contributed to the good EBF results at hospital discharge. However, integration with Primary Health Care is crucial to maintain EBF promotion and protection guidelines and actions at home, reducing the weaning rate.

The social context of the new mother, although not investigated in this study, is also a determining factor. A social support network can help sustain breastfeeding as the infants grow and new challenges arise, such as returning to work. Policies and legislation that protect breastfeeding are essential to creating a favorable and welcoming environment, promoting not only the health of postpartum women and infants but also equality in society. 

This study has some limitations that should be considered when interpreting the results. Non-probability convenience sampling may have introduced selection bias. Additionally, the inclusion criterion requiring access to Android smartphones for follow-up may have exacerbated these biases. Although these strategies were necessary to overcome the practical difficulties of accessing a random sample of preterm newborns and to increase the likelihood of contacting participants during follow-up (via app and WhatsApp), they may limit the generalizability of the results to other contexts and newborn populations. Furthermore, data collection was conducted in only two regions of the country and included only two public hospitals designated as Baby-Friendly. Given the cultural factors related to breastfeeding, it is essential to consider them, as they may further reduce the study’s generalizability. Another relevant limitation is that data collection was carried out in only two regions of the country, and exclusively in two public hospitals certified as Baby-Friendly hospital. Given the influence of cultural factors on breastfeeding practices, this study’s design may also limit the generalizability of its findings. However, it is essential to note that selecting hospitals with this certification represents a particularly relevant profile for the formulation of national public policies around breastfeeding. 

Regarding survival bias, we had a total loss of 18% during follow-up, with difficulty contacting participants as the leading cause of loss (n=42). These postpartum women may have had significant challenges with breastfeeding, and therefore, did not want to share their failure to continue breastfeeding.

## Conclusion

Most PTs were EBF at hospital discharge, with a 35.3% drop in breastfeeding 15-30 days after discharge. Factors associated with discontinuation of EBF 15-30 days after hospital discharge included maternal age >35 years; maternal education (up to elementary school; up to high school); first breast stimulation after delivery (>6 h; no stimulation); hospitalization time >14 days; age at first breastfeeding (>24 h after delivery; no breastfeeding); and EBF at hospital discharge. For weaning, only the variable of not being on EBF at hospital discharge was associated.

The results indicated that EBF indicators in premature infants were comparable or superior to those found in national and international studies, but below the targets set by international organizations for the prevention of infant and neonatal deaths.

The risk factors identified for discontinuation of EBF and weaning highlight variables that can be modified through quality care, based on scientific evidence, and involve coordinated actions between professionals and sectors that care for pregnant women and mothers of premature infants during the breastfeeding process.

Early intervention, family support, a favorable hospital environment, and supportive policies are essential to promote and maintain breastfeeding in PT after hospital discharge, ensuring care tailored to the needs of this vulnerable population.

## Data Availability

Datasets related to this article will be available upon request to the corresponding author.
