# Supplementary material for: Factors associated with the interruption of exclusive breastfeeding and weaning in premature infants after hospital discharge: a prospective cohort study
Source: Rev Lat Am Enfermagem. 2026 Mar 30;34:e4831. doi: 10.1590/1518-8345.7740.4831 (PMC13035205; doi:10.1590/1518-8345.7740.4831)
Supplement: Translation

# Translation: Fatores associados à interrupção do aleitamento materno exclusivo e desmame em prematuros no pós-alta hospitalar: estudo de coorte prospectivo^2^

**DOI:** 10.1590/1518-8345.7740.4832

**Authors:** Mariana Lamante Bueno, Júlia Carneiro Godoy de Sousa, Vinícius de Sousa Monteiro, Alinne Almeida Sousa de Sá, Ana Karina Marques Salge Mendonça, Karina Machado Siqueira, Rafael Alves Guimarães, Edilaine Giovanini Rossetto, Thaíla Corrêa Castral

**Affiliations:** 1 Universidade Federal de Goiás, Faculdade de Enfermagem, Goiânia, GO, Brasil.; 2 Bolsista da Coordenação de Aperfeiçoamento de Pessoal de Nível Superior (CAPES), Brasil.; 3 Secretaria Municipal de Saúde, Vigilância Epidemiológica de Óbitos Fetais e Infantis, Goiânia, GO, Brasil.; 4 Universidade Estadual de Londrina, Curso de Enfermagem, Londrina, PR, Brasil.

**Keywords:** Aleitamento Materno, Desmame, Recém-Nascido Prematuro, Alta do Paciente, Fatores de Risco, Enfermagem Neonatal.

## Abstract

**Objetivo:**

estimar a incidência de aleitamento materno exclusivo e desmame em prematuros e identificar fatores de risco pós-alta hospitalar.

**Método:**

coorte prospectiva com 237 participantes. Fatores associados à interrupção do aleitamento e desmame analisados por regressão de Poisson.

**Resultados:**

após a alta, 35,3% (IC95%:29,6-41,8) interromperam o aleitamento e 9,8% (IC95%:6,5-14,2) desmamaram. Associaram-se à interrupção do aleitamento: idade materna (RR=0,70; IC95%:0,49-0,99); escolaridade (fundamental: RR=1,68; IC95%:1,00-2,80; médio: RR=1,79; IC95%:1,20-2,66); estímulo das mamas (>6 h: RR=1,88; IC95%:1,13-3,13; não estimulou: RR=1,69; IC95%:0,98-2,88); hospitalização (RR=1,85; IC95%:1,10-3,08); primeira mamada (>24 h: RR=0,61; IC95%:0,38-0,99; não mamou: RR=0,66; IC95%:0,43-1,01); e aleitamento na alta (RR=3,64; IC95%:2,45-5,40). Desmame associou-se à ausência de aleitamento na alta (RR=3,64; IC95%:2,45-5,40).

**Conclusão:**

idade materna, escolaridade, hospitalização e aleitamento na alta influenciaram a interrupção do aleitamento. A ausência de aleitamento na alta elevou o risco de desmame. Intervenções precoces, suporte familiar e políticas são essenciais para manutenção do aleitamento.

## Destaques

**(1)** Monitorar a taxa de aleitamento materno em prematuros é crucial. **(2)** A incidência de aleitamento materno diminuiu após a alta hospitalar. **(3)** Conhecer os riscos para a interrupção do aleitamento auxilia políticas públicas.

## Introdução

A prematuridade é a principal causa da mortalidade neonatal^1^. Dados internacionais revelam o nascimento de 13,4 milhões de prematuros (PT) no mundo, com taxas de prematuridade entre 4% e 16% entre os países. Aproximadamente 85% dos partos PT ocorrem entre 32 e 37 semanas de gestação e, geralmente, não necessitam de cuidados intensivos^2^.

Em 2021, o Brasil registrou 2.677.101 nascidos vivos, sendo 11,3% PT, com maioria (85,9%) com idade gestacional entre 32 e 36 semanas. No Centro-Oeste, foram registrados 229.296 nascidos vivos, sendo 11,4% PT, também com a maioria (86,1%) na mesma faixa de idade^3^. 

O nascimento prematuro possui múltiplas causas (gestacionais, maternas, socioeconômicas e ambientais)^4^ ^-^ ^6^. Para 2030, uma das Metas do Desenvolvimento Sustentável é a erradicação das mortes neonatais evitáveis e a redução das mortes de recém-nascidos (RN) e natimortos^7^.

Os PT pequenos ou doentes apresentam maior risco de desenvolver doenças infecciosas, atrasos no desenvolvimento e crescimento e mortalidade na primeira infância. O aleitamento materno exclusivo (AME) surge como intervenção efetiva para prevenção desses desfechos^8^, sendo o leite humano essencial por atuar como proteção contra doenças infecciosas e crônicas, reduzir transtornos neurodesenvolvimentais e promover o vínculo mãe-filho^9^ ^-^ ^11^.

Dada a importância da amamentação e do leite humano, políticas públicas e estratégias de promoção ao AME têm sido desenvolvidas. A Iniciativa Hospital Amigo da Criança (IHAC) é uma das principais estratégias internacionais para a promoção e proteção do AME^12^. No entanto, a IHAC foi desenvolvida para RN a termo e não direciona estratégias específicas para PT. Em 2020, a Organização Mundial da Saúde (OMS) atualizou o documento, incluindo orientações adicionais para RN pequenos, doentes e/ou PT, ressaltando a presença constante da mãe e o aleitamento materno (AM)^7^.

Estudos brasileiros evidenciam que a incidência de AME em PT é de 81,4% na alta hospitalar, declinando para 75% aos 15 dias^12^ e 66,4% entre 7 e 15 dias pós-alta^13^. Essa redução progressiva reflete os desafios inerentes à amamentação de PT, associados a fatores como imaturidade fisiológica^14^, práticas hospitalares restritivas e disfunção familiar^13^.

Contudo, este cenário foi agravado pela pandemia de COVID-19, que alterou práticas de cuidado materno-neonatal, comprometendo indicadores de AM. Evidências internacionais^15^ e nacionais documentaram restrições, como proibição de contato pele a pele e exclusão de acompanhantes^16^, fatores que, somados ao apoio inadequado à amamentação e ausência de alojamento conjunto, reduziram as taxas de AME^15^.

Embora esses impactos sejam reconhecidos, há uma lacuna crítica no conhecimento sobre AME em PT no cenário pós-pandêmico, particularmente após a alta hospitalar. Estudos brasileiros existentes são anteriores à pandemia ou geograficamente limitados. 

Conhecer as taxas de AME e os fatores que facilitam ou dificultam sua manutenção é crucial para melhorar a assistência de enfermagem em unidades neonatais e sua articulação com a atenção primária. Investigar esses indicadores e as causas de interrupção do AME é fundamental para planejar políticas públicas pós-pandemia que fortaleçam o apoio ao aleitamento, permitindo monitorar ações e responder às necessidades identificadas.

O objetivo deste estudo é estimar a incidência de aleitamento materno exclusivo e desmame em prematuros e identificar fatores de risco pós-alta hospitalar.

## Método

### Delineamento do estudo

Trata-se de um estudo de coorte prospectivo. O relato do estudo seguiu as recomendações do *checklist Strengthening the Reporting of Observational Studies in Epidemiology* (STROBE)^17^. Examinou-se a incidência de AME na alta hospitalar e até 15-30 dias após a alta hospitalar, entre os prematuros expostos e não expostos aos fatores de risco (variáveis maternas e neonatais). 

### Contexto

O estudo foi realizado nas maternidades em Londrina-PR, com 24 leitos neonatais e 17 leitos de alojamento conjunto; e em uma maternidade em Goiânia-GO, com 25 leitos neonatais e 63 leitos no alojamento conjunto. As duas maternidades são referência no atendimento à gestação de alto risco e no Método Canguru (MC), oferecendo todas as etapas da linha progressiva de cuidado neonatal. Ambas possuem o título de Hospital Amigo da Criança e realizam atendimentos exclusivamente pelo Sistema Único de Saúde. A coleta de dados ocorreu entre março de 2022 e fevereiro de 2023.

### População

A população do estudo foi composta por mães e PT (< 37 semanas de idade gestacional), admitidos no alojamento conjunto e nas unidades neonatais dos dois centros.

### Critérios de elegibilidade

Os critérios de inclusão foram puérperas com filhos PT hospitalizados no alojamento conjunto, unidade de cuidado intensivo neonatal ou unidade de cuidado intermediário neonatal; que possuíam smartphone com acesso à internet, leitura e compreensão da língua portuguesa e desejo de amamentar. Os critérios de exclusão foram puérperas com contraindicação para amamentação, diagnóstico de COVID-19 ou RN com condições clínicas que impediam de receber leite humano. Foram excluídas as mães em que os RN não tinham os dados da alimentação após a alta hospitalar.

### Amostragem e amostra

Uma amostragem não probabilística por conveniência foi utilizada, método recomendado em estudos de coorte quando a população é de difícil acesso^18^, como a de RN prematuros no pós-alta. O cálculo amostral foi realizado para um estudo de coorte analítico^19^ ^-^ ^20^, utilizando os seguintes parâmetros: probabilidade de erro tipo I ou nível de significância de 5% (α=0,05), probabilidade de erro tipo II ou poder estatístico de 80% (1-β=0,2), taxa de perdas de 20% e um Risco Relativo (RR) mínimo detectável de 1,5 para representar uma diferença clinicamente significativa para as principais variáveis de exposição investigadas. Adotou-se, ainda, como parâmetro uma incidência acumulada de AME de 53,8% no grupo não exposto das principais variáveis independentes, valor obtido em estudo prévio com população similar, em que 93,8% (15/16) das variáveis independentes apresentaram incidência igual ou superior a 53,8% em grupos não expostos^13^. Por fim, aplicou-se a correção de continuidade de Fleiss no cálculo amostral^20^, resultando em uma amostra mínima estimada de 136 recém-nascidos para avaliação entre 15-30 dias após a alta hospitalar.

### Variáveis do estudo

Elegeram-se duas variáveis de desfecho: interrupção do AME e desmame 15-30 dias após a alta hospitalar. O desmame foi considerado a interrupção do AM na semana anterior ao contato com a mãe ou registro em prontuário médico.

As variáveis de exposição foram distribuídas em blocos segundo sua relação de proximidade com os desfechos, conforme abordagem teórica adaptada^13^ ^,^ ^21^. O primeiro bloco teórico (distal) incluiu as variáveis idade da mãe e escolaridade da mãe, enquanto o segundo bloco (intermediário) englobou a variável experiência prévia em amamentação. O terceiro bloco (proximal) incluiu o tipo de parto (cesárea), peso ao nascer, idade gestacional, AME na alta, tempo para estimular as mamas pela primeira vez, idade em que o lactente mamou pela primeira vez e tempo de hospitalização.

Considerou-se censura quando: 1) a mãe não respondeu às mensagens ou ligações; 2) a mãe declarou que não desejava continuar na pesquisa; 3) o lactente foi a óbito ou transferido para outro hospital. A mãe e o lactente foram excluídos do estudo apenas em caso da retirada do consentimento pela mãe. 

### Instrumentos utilizados na coleta de dados

Parte da coleta de dados foi realizada por meio do *OpenDataKit* (ODK), uma plataforma que permite o preenchimento *offline* por *smartphones* ou *tablets* e o envio dos dados para um servidor quando há conexão com a internet. Formulários de cadastro das participantes e perguntas sobre a alimentação (tipo de leite e método de alimentação) do RN no momento da alta hospitalar e após alta hospitalar foram desenvolvidos pelos pesquisadores nessa plataforma e passaram por um teste piloto. No aplicativo “AmamentaCoach”, desenvolvido por pesquisadoras da Faculdade de Enfermagem da Universidade Estadual de Londrina, formulários sobre a alimentação do RN também foram incluídos para facilitar a coleta de dados. Os dados foram exportados para o *Power BI*. 

Uma agenda de pesquisa foi elaborada em planilha *Excel* para acompanhar o seguimento das participantes. Os dados das plataformas ODK e *Power BI* foram exportados e integrados em uma planilha do *Excel*, formando um banco de dados unificado.

### Procedimento de coleta de dados

A coleta de dados foi composta de três momentos: nascimento, alta hospitalar e após a alta hospitalar. A mãe elegível foi abordada entre 72 e 120 horas após o nascimento. Após consentimento, ela foi convidada a instalar o aplicativo “AmamentaCoach” em seu dispositivo móvel.

Na alta hospitalar e entre 15 e 30 dias após a alta hospitalar, a mãe recebeu notificações pelo aplicativo para informar sobre a alta e responder perguntas sobre a alimentação do lactente. Aquelas que não responderam pelo aplicativo receberam mensagens via *WhatsApp* (três tentativas) e ligações telefônicas (três tentativas). Quando não foi possível o contato, buscaram-se as informações no prontuário eletrônico da maternidade.

### Aspectos éticos

Foram assegurados todos os aspectos éticos previstos na Resolução n.º 466/2012. O estudo foi aprovado pelo Comitê de Ética de Pesquisa (n.º 27703419.8.0000.5231). 

As mães elegíveis foram convidadas a participar do estudo e, após serem esclarecidas quanto aos objetivos, e mediante o aceite, foi feita a leitura conjunta e assinatura do Termo de Consentimento Livre e Esclarecido. No caso de mães adolescentes, o Termo de Assentimento e o Termo de Consentimento Livre e Esclarecido foram entregues ao responsável. 

### Tratamento e análise dos dados

Os dados foram importados do *Excel* e analisados no *software Stata*, versão 17.0. Foi testada a normalidade das variáveis quantitativas pelo teste Kolmogorov-Smirnov com correção de Lilliefors. Na análise descritiva, as variáveis quantitativas foram descritas como média e desvio-padrão (DP), além da mediana, percentil 25 (P25) e percentil 75 (P75) devido à ausência de normalidade. As variáveis qualitativas foram apresentadas como frequência absoluta (n) e relativa (%). Realizou-se, ainda, a análise descritiva das incidências acumuladas dos desfechos (interrupção do AME e desmame no pós-alta). 

A seguir, foi utilizada a análise bivariada, por meio da regressão de Poisson com variância robusta bivariada, para analisar a magnitude da associação entre cada variável de exposição e os desfechos. Essa estratégia foi utilizada para selecionar as variáveis para o modelo de regressão múltipla. Variáveis com valor de p<0,25 nessa análise foram incluídas em modelos de regressão múltipla^22^ ^-^ ^23^. Os resultados da análise bivariada foram apresentados como Risco Relativo Não Ajustado (RR) e respectivo intervalo de confiança de 95% (IC95%). Os resultados do modelo de regressão múltipla foram apresentados como Risco Relativo Ajustado (RRA) e respectivo IC95%, sendo considerado p< 0,05.

## Resultados

Dos 291 participantes iniciais, cinco RN foram a óbito, um RN foi transferido para outro hospital, um RN não obteve alta hospitalar, uma mãe não conseguiu baixar o *app* para coleta de dados e com duas mães não houve contato no momento da alta da UTIN. Após a alta hospitalar, não houve contato com as mães de 42 RN, e duas mães manifestaram desistência do estudo, totalizando 237 binômios mãe-filho com dados completos de alimentação 15-30 dias após a alta hospitalar como amostra final. A Tabela 1 mostra as características sociodemográficas e clínicas dos participantes. A maioria das mães tinha entre 20 e 34 anos (67,1%); 67,1% estudaram até o ensino médio e 82,7% viviam com o parceiro. Entre as gestantes, 40,5% eram primíparas, 41,4% planejaram a gravidez, 76,4% fizeram seis ou mais consultas pré-natais, 22,4% receberam informações sobre amamentação (AM) e 53,2% tinham experiência prévia com AM. Em relação ao parto, 59,9% tiveram complicações, 38,8% tiveram parto vaginal, 40,9% fizeram contato pele a pele até 72 h após o parto e 41,8% amamentaram nas primeiras 24 h.

**Tabela 1:** Características dos binômios mãe-filhos participantes da coorte do estudo (n = 237). Goiânia, GO; Londrina, PR, Brasil, 2022-2023

Variáveis	n* (%)^†^
Idade materna (anos)	
<19	28 (11,8)
20-34	159 (67,1)
>35	50(21,1)
Escolaridade materna	
Ensino fundamental incompleto ou completo	35 (14,8)
Ensino médio incompleto ou completo	159 (67,1)
Ensino superior	43 (18,1)
Reside com parceiro	196 (82,7)
Gestação planejada	98 (41,4)
Primípara	96 (40,5)
Realizou seis ou mais consultas de pré-natal	181 (76,4)
Teve intercorrência na gestação	142 (59,9)
Recebeu informação sobre aleitamento materno no pré-natal	53 (22,4)
Possui experiência prévia com amamentação	126 (53,2)
Parto vaginal	92 (38,8)
Contato pele a pele até 72 horas após parto	97 (40,9)
Primeiro estímulo nas mamas	
<6 horas após o parto	85 (35,9)
>6 horas após o parto	121 (51,1)
não estimulou até quinto dia após o parto	31 (13,1)
Peso ao nascer (gramas)	
> 2500	85 (35,9)
2500-1500	122 (51,5)
< 1500	30 (12,7)
Período em que recém-nascido mamou no peito a primeira vez	
< 24 horas após o nascimento	99 (41,8)
> 24 horas após o nascimento	51 (21,5)
não mamou no peito até o quinto dia vida	87 (36,7)
Aleitamento materno exclusivo na alta hospitalar	170 (71,7)
Idade gestacional ao nascer (semanas)	
Média (Desvio-padrão)	34,19 (2,2)
Mediana (Percentil 25-75)	35 (33,5-36)
Duração total da hospitalização (dias)	
Média (Desvio-padrão)	14,30 (18,6)
Mediana (Percentil 25-75)	6 (4-16,5)

*n = Número; ^†^% = Frequência

Os RN tinham média de 34,19 semanas gestacionais e 51,5% pesavam entre 1500 e 2500g. A maioria das mães iniciou o estímulo nas mamas após 6h do parto (51,1%). A hospitalização média dos RN foi de 14,3 dias. Nos primeiros cinco dias, 32,6% dos prematuros estavam em AME, aumentando para 71,7% na alta e reduzindo para 64,6% após 15-30 dias (Figura 1). A incidência acumulada de desmame precoce foi de 9,8% (IC95%: 6,5-14,2%).

Na análise bivariada, variáveis maternas e neonatais, como idade materna, escolaridade, experiência com aleitamento, tipo de parto, peso ao nascer e AME na alta, foram estatisticamente significativas para interrupção do AME. Para o desmame do AM, foram significativas as variáveis maternas e neonatais, como experiência com aleitamento, parto cesárea, contato pele a pele, peso ao nascer e AME na alta (Tabela 2).

**Figura 1:** Indicadores de aleitamento materno exclusivo nos primeiros cinco dias de vida, na alta hospitalar e 15-30 dias após alta hospitalar dos prematuros (n = 237). Goiânia, GO; Londrina, PR, Brasil, 2022-2023

**Tabela 2:** Taxa de incidência de interrupção de amamentação exclusiva e desmame, e Risco Relativo Bruto (RRb) em prematuros 15-30 dias após a alta hospitalar, segundo as variáveis de exposição (n = 237). Goiânia, GO; Londrina, PR, Brasil, 2022-2023

Variáveis maternas	Interrupção do AME*		RRb^†^ (IC95^‡^)	p-valor^§||^	Desmame		RRb^†^ (IC95^‡^)	p-valor^§||^
	Não (n=153)	Sim (n=84)			Não (n=214)	Sim (n=23)		
Idade (anos)								
<19	7 (25,0)	21 (75,0)	0,63 (0,32-1,23)	0,179	25 (89,3)	3 (10,7)	1,14 (0,35-3,68)	0,832
20-34	63 (39,6)	96 (60,4)	1,00		144 (90,6)	15 (9,4)	1,00	
>35	14 (28,0)	36 (72,0)	0,70 (0,44-1,15)	0,161	45 (90,0)	5 (10,0)	1,06 (0,40-2,78)	0,906
Escolaridade								
Até ensino fundamental	14 (40,0)	21 (60,0)	1,56 (0,81-3,00)	0,180	145 (88,7)	18 (11,3)	0,82 (0,14-4,64)	0,822
Até ensino médio	59 (37,1)	100 (62,9)	1,45 (0,84-2,51)	0,185	40 (93,0%)	3 (7,0)	1,62 (0,50-5,3)	0,420
Ensino superior ou mais	11 (25,6)	32 (74,4)	1,00		40 (93,0)	3 (7,0)	1,00	
Reside com parceiro								
Não	13 (31,7)	28 (68,3)	0,88(0,54-1,42)	0,592	36 (87,8)	5 (12,2)	1,33 (0,52-3,38)	0,552
Sim	71 (36,2)	125 (63,8)	1,14 (0,70-1,85)	0,592	178 (9,8)	18 (9,2)	1,00	
Realizou > 6 consultas de pré-natal								
Não	21 (37,5)	35 (62,5)	1,07 (0,73-1,60)	0,710	50 (89,3)	6 (10,7)	1,14 (0,47-2,76)	0,770
Sim	63 (34,8)	118 (65,2)	1,00		164 (90,6)	17 (9,4)	1,00	
Recebeu informação sobre aleitamento materno no pré-natal								
Não	67 (36,4)	117 (63,6)	1,13 (0,73-1,76)	0,569	164 (89,1)	20 (10,9)	1,92 (0,59-6,22)	0,277
Sim	17 (32,1)	36 (67,9)	1,00		50 (90,3)	3 (5,7)	1,00	
Experiência prévia com aleitamento materno								
Não	44 (39,6)	67 (60,4)	1,25 (0,88-1,76)	0,207	96 (86,5)	15 (13,5)	2,12 (0,93-4,83)	0,071
Sim	40 (31,7)	86 (68,3)	1,00		118 (93,7)	8 (6,3)	1,00	
Parto cesárea								
Sim	65 (44,8)	80 (55,2)	2,17 (1,40-3,37)	0,001	126 (86,9)	19 (13,1)	3,01 (1,05-8,60)	0,039
Não	19 (20,7)	73 (79,3)	1,00		88 (95,7)	4 (4,3)	1,00	
Contato pele a pele até 72 horas após parto								
Não	52 (37,1)	88 (62,9)	1,12 (0,79-1,61)	0,515	122 (87,1)	18 (12,9)	2,49 (0,95-6,50)	0,062
Sim	32 (33)	65 (67)	1,00		92 (94,8)	5 (5,2)	1,00	
Primeiro estímulo nas mamas (horas)								
<6 h após o parto	15 (17,6)	70 (82,4)	1,00		83 (97,6)	2 (2,4)	1,00	
>6 h após o parto	52 (43,0)	69 (57,0)	2,43 (1,47-4,03)	0,001	109 (90,1)	12 (9,9)	4,21 (0,96-18,40)	0,056
Não estimulou até 5 dias após o parto	17 (54,8)	14 (45,2)	3,10 (1,77-5,44)	<0,001	22 (71,0)	9 (29,0)	12,3 (2,81-54,15)	0,001
**variáveis neonatais**	Interrupção do AME*		**RRb^†^ (IC95^‡^)**	**p-valor^§||^**	**Desmame**		**RRb^†^ (IC95^‡^)**	**p-valor^§||^**
	**Não (n=153)**	**Sim (n=84)**			**Não (n=214)**	**Sim (n=23)**		
Peso ao nascer (gramas)								
> 2.500	19 (22,4)	66 (77,6)	1,00		81 (95,3)	4 (4,7)	0,71 (0,22-2,31)	0,578
2.500-1.500	43 (35,2)	79 (64,8)	1,58 (0,99-2,50)	0,055	114 (93,4)	8 (6,6)	1,00	
< 1.500	22 (73,3)	8 (26,7)	3,28 (2,08-5,15)	<0,001	19 (63,3)	11 (36,7)	5,59 (2,46-12,70)	<0,001
Idade gestacional ao nascer (semanas)								
<28	1 (20)	4 (80)	0,61 (0,10-3,58)	0,585	4 (80)	1 (20)	3,06 (0,49-19,03)	0,231
28-32	13 (72,2)	5 (27,8)	2,20 (1,56-3,11)	<0,001	10 (55,6)	8 (44,4)	6,79 (3,29-14,02)	<0,001
32-37	70 (32,7)	144 (67,3)	1,00		200 (93,5)	14 (6,5)	1,00	
Tempo de hospitalização (dias)								
< 7	28 (23,3)	92 (76,7)	1,00		117 (97,5)	3 (2,5)	1,00	
7-14	13 (24,5)	40 (75,5)	1,05 (0,59-1,87)	0,865	50 (94,3)	3 (5,7)	2,26 (0,47-10,9)	0,308
> 14	43 (67,2)	21 (32,8)	2,88 (1,99-4,16)	<0,001	47 (73,4)	17 (26,6)	10,62 (3,22-35,0)	<0,001
Idade em que recém-nascido mamou a primeira vez no peito								
< 24 horas após o parto	28 (28,3)	71 (71,7)	1,00		96 (97)	3 (3)	1,00	
> 24 horas após o parto	16 (31,4)	35 (68,6)	1,11 (0,66-1,85)	0,693	46 (90,2)	5 (9,8)	3,23 (0,80-13,03)	0,099
Não mamou no peito até o quinto dia de vida	40 (46,0)	47 (54,0)	1,62 (1,10-2,40)	0,014	72 (82,8)	15 (17,2)	5,68 (1,70-19,04)	0,005
Estava em AME* na alta hospitalar								
Não	55 (82,1)	12 (17,9)	4,81 (3,39-6,83)	<0,001	48 (71,6)	19 (28,4)	12,05 (4,24-34,19)	<0,001
Sim			1,00		166 (97,6)	4 (2,4)	1,00	

*AME = Aleitamento materno exclusivo; ^†^RRb = Risco relativo não ajustado; ^‡^CI95% = Intervalo de confiança de 95%; ^§^p=0,05; ^||^Teste de qui-quadrado de Wald

Na análise múltipla hierárquica (Tabela 3), idade materna > 35 anos reduziu o risco de interrupção do AME em 0,30 vezes. A baixa escolaridade aumentou o risco: 1,68 vezes para até ensino fundamental e 1,79 vezes para até ensino médio. Estímulo das mamas após 6h ou ausência de estímulo aumentou o risco em 1,88 e 1,69 vezes, respectivamente. O tempo de hospitalização aumentou o risco em 1,85 vezes. Primeira mamada após 6h aumentou o risco em 1,61 vezes, e ausência de mamada até o quinto dia, em 1,66 vezes. Não estar em AME na alta aumentou o risco em 3,64 vezes. 

Na análise multivariada hierárquica, não estar em AME na alta hospitalar aumentou em 6,13 vezes o risco para desmame do AM 15-30 dias após a alta, conforme demonstrado na Tabela 4.

**Tabela 3:** Análise multivariada por meio de regressão de Poisson com modelo hierárquico para interrupção do aleitamento materno exclusivo 15-30 dias após a alta hospitalar dos prematuros e fatores associados (n = 237). Goiânia, GO; Londrina, PR, Brasil, 2022-2023

Variáveis	Modelo I		Modelo II		Modelo III	
	RRA*	IC95^†^	RRA*	IC95^†^	RRA*	IC95^†^
Bloco 1						
Idade da mãe (anos)						
< 19	0,57 (0,28-1,14)		0,50 (0,25-1,02)		0,63 (0,36-1,12)	
20-34	1,00		1,00		1,00	
> 35	0,71 (0,43-1,16)		0,75 (0,45-1,25)		0,70 (0,50-1,0)^‡^	
Escolaridade da mãe						
Até ensino fundamental	1,75 (0,90-3,40)		1,95 (1,0-3,7)^||^		1,68 (1,00-2,80)^‡^	
Até ensino médio	1,50 (0,85-2,62)		1,6 (0,9-2,7)		1,79 (1,20-2,66)^‡^	
Ensino superior ou mais	1,00		1,00		1,00	
**Bloco 2**						
Experiência prévia com aleitamento materno						
Não			1,38 (1,0-1,9)		1,26 (0,94-1,69)	
Sim			1,00		1,00	
**Bloco 3**						
Parto cesárea						
Não					1,00	
Sim					1,26 (0,85-1,88)	
Primeiro estímulo das mamas						
< 6 h após parto					1,00	
>6 h após parto					1,88 (1,13-3,13)^‡^	
não estimulou até quinto dia após o parto					1,69 (0,98-2,88)^‡^	
Peso ao nascer (gramas)						
> 2.500					1,00	
2.500-1.500					1,24 (0,72-2,14)	
< 1.500				0,89 (0,61-1,28)	
Idade gestacional ao nascer (semanas)						
< 28					0,37 (0,10-1,32)	
28-32					0,99 (0,71-1,37)	
32-37					1,00	
Tempo de hospitalização						
< 7 dias					1,00	
7-14 dias					0,97 (0,58-1,63)	
> 14 dias					1,85 (1,10-3,08)^‡^	
Idade em que mamou pela primeira vez no peito						
< 24 horas após o parto					1,00	
> 24 horas após o parto					0,61 (0,38-0,99)^‡^	
não mamou no peito até o quinto dia de vida					0,66 (0,43-1,01)^‡|^	
Aleitamento materno exclusivo na alta hospitalar						
Não					3,64 (2,45-5,40)^§^	
Sim					1,00	

*RRa = Risco Relativo ajustado; ^†^IC95% = Intervalo de confiança de 95%; ^‡^p<0,05 Teste de qui-quadrado de Wald; ^§^p<0,001

**Tabela 4:** Análise multivariada por meio regressão de Poisson com modelo hierárquico para desmame do aleitamento materno 15-30 dias após a alta hospitalar dos prematuros e fatores associados (n = 237). Goiânia, GO; Londrina, PR, Brasil, 2022-2023

Variáveis	Distal		Intermediário		Proximal	
	RRA*	IC95%^†^	RRA*	IC95^†^	RRA*	IC95^†^
Bloco 1						
Experiência prévia com aleitamento materno						
Não			2,13 (0,93-4,84)		1,97 (0,96-4,02)	
Sim			1,00		1,00	
**Bloco 2**						
Parto cesárea						
Não					1,00	
Sim					1,23 (0,42-3,56)	
Primeiro estímulo das mamas						
< 6 horas após o parto					1,00	
>6 horas após o parto					1,75 (0,44-6,89)	
Não estimulou até o quinto dia após o parto					2,47 (0,62-9,83)	
Peso ao nascer (gramas)						
> 2500				1,00	
< 1500				1,50 (0,55-4,07)	
> 2500				2,72 (0,64-11,4)	
Idade gestacional ao nascer (semanas)						
< 28				0,85 (0,19-3,77)	
28-32				1,84 (0,68-4,96)	
32-37				1,00	
Tempo de hospitalização (dias)						
< 7				1,00	
7-14				1,85 (0,36-9,35)	
> 14				2,98 (0,64-13,72)	
Contato pele a pele até 72 horas após parto						
Não				1,50 (0,51-4,35)	
Sim				1,00	
Aleitamento materno exclusivo na alta hospitalar						
Não				6,13 (2,43-15,40)^‡^	
Sim				1,00	

*RRa = Risco Relativo ajustado, ^†^IC95% = Intervalo de confiança de 95%, ^‡^p<0,001 Teste de qui-quadrado de Wald

## Discussão

Os benefícios do AM são diversos, tanto para a mãe/família quanto para o lactente^8^ ^-^ ^10^. Para o grupo dos PT, os benefícios se estendem e podem impactar diretamente a resposta clínica desse RN internado em unidade neonatal, reduzindo infecções, entre outras complicações, além de o leite humano ser essencial para o desenvolvimento do PT. São muitas as dificuldades para manter esse lactente em AM e elas se estendem após a alta hospitalar^24^.

Neste estudo, observou-se que houve redução na incidência de AME após a alta hospitalar (de 71,7% para 64,2%). Estudos em outras cidades e países mostram diferenças nos indicadores de AME na alta e após a alta hospitalar, destacando a importância de compreender o contexto e os fatores de risco ao comparar esses dados.

Estudo em Goiânia-GO, com 113 PT, também mostrou uma queda na incidência de AME 7-15 dias após a alta (de 81,4% para 66,4%)^13^. Em Recife-PE, um estudo transversal em dois hospitais públicos com 108 PT encontrou uma incidência de AME de 85,2% na alta, 75% aos 15 dias e 46,3% aos 30 dias^25^. Estudo de coorte, no Rio de Janeiro-RJ, acompanhou 1.003 lactentes (22,5% eram PT). A prevalência de AME na alta hospitalar foi de apenas 14,3% entre os PT^24^. 

Após a alta hospitalar, o índice de AME foi menor que o encontrado no estudo transversal conduzido em Recife com 108 PT^25^ e no estudo de coorte prospectivo conduzido em Goiânia com 113 PT^13^ 15-30 dias após a alta; contudo, foi maior em relação ao estudo de Recife^25^ com 30 dias após a alta hospitalar. Todos os estudos foram realizados em hospitais Amigo da Criança, sendo que a coleta de dados em nosso estudo ocorreu durante a pandemia de COVID-19. 

Na Grécia, um estudo retrospectivo com 279 mães e RN, sendo 122 (43,7%) PT (15,4% com IG < 32 semanas e 28,3% PT tardios), mostrou uma prevalência de AME de 58,1% no primeiro mês. A duração média do AME foi de 2 meses nos PT e 3 meses nos bebês a termo (p=0,002). Lactentes prematuros tiveram 1,64% maior risco de interromper o AME^26^. Em Xangai (China), um estudo de coorte com 500 PT mostrou que 19% eram amamentados um mês após a alta. Durante a internação na unidade neonatal, as mães não podiam amamentar diretamente, e a ordenha do leite humano era administrada pelos profissionais^27^.

Na Austrália, um estudo de coorte com 270 PT tardios mostrou que 74% dos bebês tiveram alta em AME, reduzindo para 41% com 6 semanas de idade corrigida^28^. Na Suécia, um estudo com 29.445 PT (6,6% extremos, 16% muito prematuros e 18% moderados) verificou uma incidência de AME de 59% em 2004 e 45% em 2013^29^.

Observou-se que a incidência de AME na alta^28^ ^-^ ^29^ e após a alta^26^ ^-^ ^28^ foi maior em nosso estudo em comparação a outros países. Apesar das diferenças, os índices de AME diminuem expressivamente após a alta hospitalar, apontando os desafios para manutenção do AME nesta população, mesmo em países que possuem política de incentivo à amamentação, como é o exemplo do Brasil e da Suécia.

Esses números, embora expressivos, ainda estão abaixo do preconizado pela OMS, que recomenda 90% na alta e 70% de AME nos primeiros 6 meses^30^. Contudo, não existem especificações destas metas para os PT, mesmo com evidências de uma maior vulnerabilidade desses bebês para o desmame precoce.

Dados globais revelaram que, entre 2015 e 2021, apenas 48% dos lactentes menores de seis meses foram amamentados exclusivamente no mundo, e 16% das mortes infantis anuais ocorrem devido ao aleitamento inadequado^30^ ^).^

Uma iniciativa do Fundo das Nações Unidas para a Infância (UNICEF) e OMS identificou prioridades para os países protegerem e promoverem a amamentação. Apenas 16% dos países alinharam sua legislação ao Código Internacional de Substituto do Leite Materno, com África e Ásia na liderança. Apenas 9% dos países atendem à recomendação da licença-maternidade remunerada, com a Oceania não atendendo em 31%. Em 14% dos países, a maioria dos nascimentos ocorreu em Hospitais Amigo da Criança; 70% tinham programas comunitários de aleitamento; 23% possuíam suporte em emergências; e 43% coletaram dados sobre AME nos últimos cinco anos^30^. No *ranking World Breastfeeding Trends Initiative*, o Brasil está na 18ª posição, liderado por Bangladesh (1ª posição)^31^.

A melhoria desses indicadores só é possível se forem identificados os fatores de risco para o desmame e políticas e ações específicas para cada contexto forem planejadas. Neste estudo, a análise multivariada revelou a baixa escolaridade materna como fator de risco e a idade materna como fator de proteção para a interrupção do AME 15-30 dias após a alta hospitalar do PT. Um estudo em Maceió-AL com 161 PT em AME na alta hospitalar encontrou resultado semelhante, sugerindo que mulheres nessa faixa etária têm maior conscientização sobre os benefícios da AME, apesar dos riscos obstétricos associados^32^. Outro estudo com 622 puérperas em Porto Alegre-RS associou a idade materna ≥ 35 anos como fator de proteção ao AME. Por outro lado, mulheres entre 26 e 34 anos enfrentam desafios relacionados à vida laboral, como a necessidade de retornar ao trabalho, licença-maternidade curta e insegurança financeira^33^.

Uma revisão sistemática identificou 36 fatores associados à interrupção do AME, sendo as variáveis socioeconômicas, como local de residência, escolaridade e raça, consideradas fatores distais. A escolaridade materna foi a variável mais investigada, com a baixa escolaridade frequentemente associada à interrupção do AME^21^. Em nosso estudo, a baixa escolaridade materna também foi associada ao maior risco de interrupção do AME 15-30 dias após a alta hospitalar. No entanto, investigamos apenas duas variáveis distais (escolaridade e idade da mãe), o que foi uma limitação do estudo.

A baixa escolaridade pode levar à dificuldade de compreensão das orientações e de manutenção do AME^34^. Além disso, mães com mais anos de estudo possivelmente têm maior controle no ambiente de trabalho, recebendo mais apoio e incentivo ao AME por mais tempo^35^.

Ações educativas para melhorar a compreensão sobre amamentação são efetivas para o início e manutenção do AME, especialmente se realizadas durante a gestação em unidades básicas de saúde ou ambulatórios de risco. Dois estudos mostraram aumento do conhecimento após ações educativas (exposições dialogadas, vídeos, cartazes), mas não analisaram a melhoria nas taxas de AME^36^ ^-^ ^37^.

A análise multivariada revelou associações com várias variáveis proximais: tempo de estímulo das mamas pela primeira vez, idade do primeiro aleitamento, tempo de hospitalização e AME na alta hospitalar. A ausência de AME na alta foi a variável de maior risco para a interrupção do AME (3,64 vezes maior) e para o desmame (6,13 vezes maior) após a alta. 

Um estudo de coorte com 12 países (Albânia, Brasil, Bulgária, Chipre, Chile, Grécia, Israel, Malta, Portugal, Espanha, Turquia e Reino Unido) investigou o impacto da pandemia nos fatores que interferem nas taxas de amamentação, analisando dados de 5.612 mulheres de países de renda alta e média-alta. Fatores de risco para o AME incluíram primiparidade, idade do bebê, prematuridade, internação em Unidade de Terapia Intensiva Neonatal (UTIN), falta de apoio à amamentação no pré-natal e após a alta, e tratamento psiquiátrico^38^.

Outros estudos também encontraram associação entre interrupção do AME em PT e tempo de hospitalização^24^ ^,^ ^39^. Em nosso estudo, a maioria dos bebês ficou menos de 15 dias hospitalizada. Estudos longitudinais com maior tempo de seguimento poderão esclarecer melhor essa relação.

Uma política nacional efetiva e de baixo custo que contribui para a redução do tempo de hospitalização é o Método Canguru (MC), sendo uma estratégia prioritária para bebês PT e de baixo peso. Segundo o Ministério da Saúde, um dos critérios para alta hospitalar na segunda etapa do MC é o AME. O MC reduz o tempo de separação entre o RN e os pais, facilita o vínculo familiar, auxilia na estabilização clínica do RN e garante o estímulo e a manutenção da amamentação. Após a alta, esses bebês recebem acompanhamento especializado pela equipe de uma Unidade Básica de Saúde, em parceria com a maternidade de origem, durante as primeiras semanas em casa^40^.

Há evidências de que a amamentação precoce e o contato pele a pele em PT^26^ e a termo^41^ contribuem para a manutenção do AME. Neste estudo, menos da metade dos PT realizaram o MC até 72 horas após o parto. Destaca-se que apenas uma UTIN participante possui um protocolo baseado em evidências com critérios para o início da posição canguru, geralmente subjetivo, atrelado à estabilidade clínica do bebê, o que pode postergar o início deste contato.

Alguns estudos associaram o tipo de alimentação do PT na UTIN e a oferta de leite humano com a duração do AME^26^ ^-^ ^27^ ^,^ ^41^, mas não encontramos estudos sobre a idade em que o PT mamou pela primeira vez associada à interrupção do AME. Evidências científicas indicam que a estabilidade do bebê deve ser o único critério para início da amamentação, pois PT são competentes para amamentar precocemente^42^. Em nosso estudo, 87 (36,7%) dos PT não mamaram no peito até o quinto dia de vida.

A experiência prévia com a amamentação foi o único fator intermediário na análise multivariada hierárquica. Metade das puérperas (53,2%) teve experiência anterior com amamentação. Essa variável mostrou associação com a interrupção do AME na análise bivariada, mas não foi significativa na análise multivariada, ao contrário de outros estudos^26^ ^,^ ^41^. 

Uma revisão sistemática de 15 estudos com 16.579 mães revelou que a experiência prévia com amamentação está associada ao início e à duração da amamentação subsequente. A qualidade dessa experiência, se satisfatória e prolongada, influenciou positivamente a duração da amamentação^43^. Estudos futuros devem investigar a qualidade e a duração da experiência anterior com a amamentação, como fatores de risco da continuidade do AME.

A maioria das mães realizou o primeiro estímulo nas mamas após 6 horas do parto (51,1%) ou não o fez até a entrevista (13,1%). Estudo com 263 mães de PT encontrou maior risco de ausência de AME na alta hospitalar quando a ordenha começou após 48 horas^39^. Um estudo investigou os fatores associados ao início tardio da ordenha da mama (> 6 horas após o parto) em 129 mães de PT. Experiência anterior em UTIN, sexo masculino do bebê, bem-estar psicológico materno comprometido, cesariana e idade gestacional ao nascimento foram identificados como fatores de risco para a ordenha tardia^44^.

Recomenda-se que as orientações e os cuidados adequados às puérperas para ordenha do leite sejam realizados ainda na gestação e nas primeiras 3-6 horas após o parto, ou o mais rápido possível^7^ ^,^ ^45^. A mãe deve receber instruções e demonstrações de como ordenhar o leite manualmente ou por bomba^42^. A alimentação com leite humano impacta significativamente a colonização intestinal precoce do PT, protegendo contra a enterocolite necrosante^45^.

O planejamento e a articulação das ações entre os setores hospitalares são essenciais para garantir o início precoce da ordenha e facilitar a oferta eficiente de leite humano ao PT. Quanto ao desmame do AM 15-30 dias após a alta hospitalar do PT, a análise multivariada hierárquica identificou apenas uma variável de risco significativa: não estar em AME na alta hospitalar (risco 6,13 vezes maior). Estudos anteriores corroboram que a ausência de AME na alta está associada ao aumento do risco de desmame precoce^25^ ^,^ ^46^. A oferta de leite humano na primeira alimentação enteral do PT, quando feita de forma suplementar durante a hospitalização, foi associada ao desmame após a alta hospitalar em outros estudos, uma vez que, ao ser prescrito no período de internação, as puérperas questionavam sua capacidade de amamentar^24^ ^,^ ^26^ ^-^ ^27^.

Diversos obstáculos após a alta hospitalar podem dificultar a manutenção do AME, como a adaptação à rotina domiciliar, percepção materna de pouco leite, falta de apoio familiar e profissional, retorno ao trabalho precoce, introdução precoce de alimentos complementares e falta de informação sobre a importância do AME^24^ ^-^ ^25^ ^,^ ^41^. Estudos futuros que acompanhem esses prematuros por maior tempo após a alta são importantes para investigar outros fatores que interferem no desmame precoce.

Os profissionais de saúde que atuam em unidades neonatais devem implementar intervenções individualizadas para promover o desenvolvimento do PT, protegendo e incentivando a amamentação desde o início, facilitando o contato pele a pele precoce e promovendo sua continuidade^47^.

Entendemos que as diferenças culturais e as políticas de saúde influenciam os desfechos da amamentação de prematuros, integrando evidências internacionais e experiências locais, a exemplo do Método Canguru no Brasil e da variabilidade na prática do contato pele a pele. Tais disparidades revelam um cenário complexo de desigualdade nas taxas AME. Diante disso, os autores defendem a urgência de pesquisas longitudinais e prospectivas em enfermagem, voltadas à avaliação do impacto de fatores culturais, sistêmicos e das práticas assistenciais sobre a duração do AME após a alta. Também destacam a importância de investigar como a experiência prévia de amamentação e o contexto assistencial afetam as decisões maternas, além de testar intervenções de enfermagem cientificamente embasadas e culturalmente adaptadas.

Os resultados deste estudo reforçam que a implementação de ações baseadas em evidências científicas e políticas existentes pode melhorar os indicadores de AME e reduzir o desmame precoce após a alta hospitalar do PT, favorecendo a AME até os seis meses de idade.

O papel crucial do enfermeiro na liderança do cuidado integral ao PT e sua família, tanto na UTIN quanto após a alta hospitalar, é destacado. O planejamento e a execução de ações para promoção da amamentação são responsabilidades fundamentais desses profissionais^47^.

A assistência aos PT em hospitais com título de Amigo da Criança e implementação do MC provavelmente contribuiu para os bons resultados de AME na alta hospitalar. Entretanto, a integração com a Atenção Básica de Saúde é crucial para manter as orientações e ações de promoção e proteção do AME em casa, reduzindo sua taxa de desmame.

O contexto social da puérpera, embora não investigado neste estudo, também é determinante. A rede de apoio social pode facilitar a continuidade da amamentação conforme o lactente cresce e novos desafios surgem, como o retorno ao trabalho. Políticas e legislações que protejam a amamentação são essenciais para criar um ambiente favorável e acolhedor, promovendo não apenas a saúde de puérperas e lactentes, mas a igualdade na sociedade.

Este estudo apresenta algumas limitações que devem ser consideradas na interpretação dos resultados. A amostragem não probabilística por conveniência pode ter introduzido viés de seleção. Adicionalmente, o critério de inclusão que exigia acesso a smartphones com sistema Android para acompanhamento pode ter potencializado esses vieses. Embora essas estratégias tenham sido necessárias para superar as dificuldades práticas do acesso a uma população aleatória de RN prematuros e ampliar as possibilidades de viabilizar o contato com as participantes durante o seguimento (via aplicativo e *WhatsApp*), elas podem limitar a generalização dos resultados para outros contextos e populações de RN. Além disso, a coleta de dados ocorreu em apenas duas regiões do país e incluiu apenas dois hospitais públicos e com o título de Amigo da Criança. Visto o componente cultural relacionado ao aleitamento materno, é importante considerar também esses fatores que podem reduzir ainda mais a generalização do estudo. Outra limitação relevante refere-se ao fato de a coleta de dados ter sido realizada em apenas duas regiões do país, incluindo exclusivamente dois hospitais públicos certificados como Amigos da Criança. Considerando a influência de fatores culturais nas práticas de aleitamento materno, essa característica do estudo também pode restringir a generalização dos achados. Entretanto, é importante destacar que a seleção de hospitais com essa certificação representa um perfil particularmente relevante para a formulação de políticas públicas nacionais na área de aleitamento materno.

Quanto ao viés de sobrevivência, tivemos uma perda total de 18% no seguimento, sendo a dificuldade de contato a maior causa das perdas (n=42). É possível que essas puérperas tivessem maiores dificuldades no aleitamento materno e, por isso, não quisessem compartilhar o seu fracasso na descontinuidade do aleitamento materno. 

## Conclusão

A maioria dos PT estava em AME na alta hospitalar, com uma queda de 35,3% após 15-30 dias da alta hospitalar. Os fatores associados à interrupção do AME 15-30 dias após a alta hospitalar incluíram idade materna >35 anos; escolaridade materna (até ensino fundamental; até ensino médio); primeiro estímulo das mamas após o parto (>6 h; não estimulou); tempo de hospitalização >14 dias; idade que mamou pela primeira vez no peito (>24 h após o parto; não mamou no peito); e AME na alta hospitalar. Para o desmame, apenas a variável não estar em AME na alta hospitalar mostrou associação.

Os resultados indicaram que os indicadores de AME em lactentes prematuros foram comparáveis ou superiores aos encontrados em estudos nacionais e internacionais, porém abaixo das metas estabelecidas por organizações internacionais para a prevenção de mortes infantis e neonatais.

Os fatores de risco identificados para a interrupção do AME e o desmame destacam variáveis passíveis de modificação com assistência de qualidade baseada em evidências científicas, envolvendo ações articuladas entre profissionais e setores que atendem gestantes e mães de PT durante o processo de amamentação.

A intervenção precoce, suporte familiar, ambiente hospitalar favorável e políticas de apoio são essenciais para promover e manter o aleitamento materno em PT após a alta hospitalar, garantindo cuidados adaptados às necessidades dessa população vulnerável.

## Declaração de Disponibilidade de Dados:

Os conjuntos de dados relacionados a este artigo estarão disponíveis mediante solicitação ao autor correspondente.

# Translation: Factores asociados con la interrupción de la lactancia materna exclusiva y el destete en bebés prematuros tras el alta hospitalaria: estudio prospectivo de cohortes^3^

**DOI:** 10.1590/1518-8345.7740.4830

**Authors:** Mariana Lamante Bueno, Júlia Carneiro Godoy de Sousa, Vinícius de Sousa Monteiro, Alinne Almeida Sousa de Sá, Ana Karina Marques Salge Mendonça, Karina Machado Siqueira, Rafael Alves Guimarães, Edilaine Giovanini Rossetto, Thaíla Corrêa Castral

**Affiliations:** 1 Universidade Federal de Goiás, Faculdade de Enfermagem, Goiânia, GO, Brasil.; 2 Becaria (o) de la Coordenação de Aperfeiçoamento de Pessoal de Nível Superior (CAPES), Brasil.; 3 Secretaria Municipal de Saúde, Vigilância Epidemiológica de Óbitos Fetais e Infantis, Goiânia, GO, Brasil.; 4 Universidade Estadual de Londrina, Curso de Enfermagem, Londrina, PR, Brasil.

**Keywords:** Lactancia Materna, Destete, Recien Nacido Prematuro, Alta del Paciente, Factores de Riesgo, Enfermería Neonatal.

## Abstract

**Objetivo:**

estimar la incidencia de lactancia materna exclusiva y destete en bebés prematuros e identificar factores de riesgo tras el alta hospitalaria.

**Método:**

estudio de cohorte prospectivo con 237 participantes. Factores asociados a la interrupción de la lactancia y el destete analizados por regresión de Poisson.

**Resultados:**

tras el alta, el 35,3% (IC 95%: 29,6-41,8) interrumpió la lactancia materna y el 9,8% (IC 95%: 6,5-14,2) se destetó. Los siguientes se asociaron con la interrupción de la lactancia materna: edad materna (RR=0,70; IC 95%: 0,49-0,99); Escolaridad (primaria: RR=1,68; IC 95%: 1,00-2,80; medio: RR=1,79; IC 95%: 1,20-2,66); estimulación mamaria (>6 h: RR=1,88; IC 95%: 1,13-3,13; no estimuló: RR=1,69; IC 95%: 0,98-2,88); hospitalización (RR=1,85; IC 95%: 1,10-3,08); primera alimentación (>24 h: RR=0,61; IC 95%: 0,38-0,99; no amamantó: RR=0,66; IC 95%: 0,43-1,01); y lactancia materna al alta (RR=3,64; IC 95%: 2,45-5,40). El destete se asoció con la ausencia de lactancia al momento del alta (RR=3,64; IC 95%: 2,45-5,40).

**Conclusión:**

la edad materna, la escolarización, la hospitalización y la lactancia al momento del alta influyeron en la interrupción de la lactancia. La ausencia de lactancia materna al momento del alta aumentó el riesgo de destete. Las intervenciones tempranas, el apoyo familiar y las políticas son esenciales para mantener la lactancia materna.

## Destacados

**(1)** Controlar la tasa de lactancia materna en bebés prematuros es fundamental. **(2)** La incidencia de lactancia materna disminuyó tras el alta hospitalaria. **(3)** Conocer los riesgos de la interrupción de la lactancia materna ayuda a las políticas públicas.

## Introducción

La prematuridad es la principal causa de mortalidad neonatal^1^. Los datos internacionales revelan el nacimiento de 13,4 millones de prematuros (PT) en el mundo, con tasas de prematuridad entre el 4% y el 16% entre países. Aproximadamente el 85% de los partos de PT ocurren entre las semanas 32 y 37 de gestación y generalmente no requieren cuidados intensivos^2^.

En 2021, Brasil registró 2.677.101 nacimientos vivos, el 11,3% de los cuales fueron PT, y la mayoría (85,9%) con una edad gestacional entre 32 y 36 semanas. En el Medio Oeste se registraron 229.296 nacidos vivos, de los cuales el 11,4% eran PT, y la mayoría (86,1%) también pertenecían al mismo grupo de edad^3^.

El nacimiento prematuro tiene múltiples causas (gestacionales, maternas, socioeconómicas y ambientales)^4^ ^-^ ^6^. Para 2030, uno de los Objetivos de Desarrollo Sostenible es la erradicación de las muertes neonatales prevenibles y la reducción de las muertes neonatales (RN) y de los nacimientos muertos^7^.

Los PT pequeños o enfermos tienen un mayor riesgo de desarrollar enfermedades infecciosas, retrasos en el desarrollo y el crecimiento, y mortalidad en la primera infancia. La lactancia materna exclusiva (LME) se presenta como una intervención eficaz para prevenir estos resultados^8^, ya que la leche materna es esencial porque actúa como protección contra enfermedades infecciosas y crónicas, reduce los trastornos del desarrollo neurológico y promueve el vínculo madre-hijo^9^ ^-^ ^11^.

Dada la importancia de la lactancia y la leche maternas, se han desarrollado políticas públicas y estrategias para promover la LME. La Iniciativa Hospitalaria Amiga del Niño (IHAC) es una de las principales estrategias internacionales para la promoción y protección de la LME^12^. Sin embargo, la IHAC se desarrolló para RN a término completo y no dirige estrategias específicas para los PT. En 2020, la Organización Mundial de la Salud (OMS) actualizó el documento, incluyendo directrices adicionales para RN pequeños, enfermos y/o PT, enfatizando la presencia constante de la madre y la lactancia materna (LM)^7^.

Los estudios brasileños muestran que la incidencia de LME en el PT es del 81,4% en el alta hospitalaria, disminuyendo al 75% a los 15 días^12^ y al 66,4% entre 7 y 15 días después del alta^13^. Esta reducción progresiva refleja los desafíos inherentes a la lactancia materna en PT, asociados a factores como la inmadurez fisiológica^14^, las prácticas hospitalarias restrictivas y la disfunción familiar^13^.

Sin embargo, este escenario se agravó con la pandemia de COVID-19, que cambió las prácticas de cuidado materno-neonatal, comprometiendo los indicadores de la LM. Evidencias internacionales^15^ y nacionales documentaron restricciones como la prohibición del contacto piel con piel y la exclusión de acompañantes^16^, factores que, sumados a un apoyo insuficiente a la lactancia materna y la ausencia de alojamiento conjunto, redujeron las tasas de LME^15^.

Aunque estos impactos son reconocidos, existe una brecha crítica en el conocimiento sobre la LME en PT en el contexto pospandemia, especialmente tras el alta hospitalaria. Los estudios brasileños existentes son previos a la pandemia o geográficamente limitados.

Conocer las tasas de LME y los factores que facilitan o dificultan su mantenimiento es fundamental para mejorar la atención de enfermería en las unidades neonatales y su articulación con la atención primaria. Investigar estos indicadores y las causas de la interrupción de la LME es esencial para planificar políticas públicas post-pandemia que refuercen el apoyo a la lactancia materna, permitiendo monitorizar las acciones y responder a las necesidades identificadas.

El objetivo de este estudio es estimar la incidencia de la lactancia materna exclusiva y el destete en bebés prematuros e identificar factores de riesgo tras el alta hospitalaria.

## Método

### Diseño del estudio

Este es un estudio de cohorte prospectivo. El informe del estudio siguió las recomendaciones de la lista de verificación *Strengthening the Reporting of Observational Studies in Epidemiology* (STROBE)^17^. Se examinó la incidencia de LME al alta hospitalaria y hasta 15-30 días después del alta, entre bebés prematuros expuestos y no expuestos a factores de riesgo (variables maternas y neonatales).

### Contexto

El estudio se llevó a cabo en hospitales de maternidad en Londrina-PR, con 24 camas neonatales y 17 camas con alojamiento; y en un hospital de maternidad en Goiânia-GO, con 25 camas neonatales y 63 camas en alojamiento interno. Los dos hospitales de maternidad son una referencia en el cuidado de embarazos de alto riesgo y en el Método Canguro (MC), ofreciendo todas las etapas de la línea progresiva de atención neonatal. Ambos tienen el título de *Hospital Amigo da Criança* (Hospital Amigo del Niño) y ofrecen atención exclusivamente a través del Sistema Unificado de Salud. La recopilación de datos tuvo lugar entre marzo de 2022 y febrero de 2023.

### Población

La población del estudio estaba compuesta por madres y PT (< 37 semanas de edad gestacional), ingresadas en las unidades de alojamiento y neonatales de los dos centros.

### Criterios de elegibilidad

Los criterios de inclusión fueron mujeres puérperas con hijos PT hospitalizados en alojamiento conjunto, unidad de cuidados intensivos neonatales o unidad de cuidados intermedios neonatales; que tuvieran un *smartphone* con acceso a Internet, capacidad para leer y comprender el portugués y deseo de amamantar. Los criterios de exclusión fueron mujeres puérperas con contraindicaciones para la lactancia materna, diagnóstico de COVID-19 o RN con condiciones clínicas que les impedían recibir leche materna. Se excluyeron las madres en las que el RN no tenía datos de alimentación tras el alta hospitalaria.

### El muestreo y muestra

Se utilizó un muestreo por conveniencia no probabilístico, un método recomendado en estudios de cohorte cuando la población es de difícil acceso^18^, como la de los RN prematuros en el periodo posterior al alta. El tamaño de la muestra se calculó para un estudio analítico de cohorte^19^ ^-^ ^20^, utilizando los siguientes parámetros: probabilidad de error tipo I o nivel de significación del 5% (α=0,05), probabilidad de error o poder estadístico tipo II del 80% (1-β=0,2), tasa de pérdida del 20,0% y un Riesgo Relativo (RR) mínimo detectable de 1,5 para representar una diferencia clínicamente significativa para las principales variables de exposición investigadas. También se adoptó como parámetro una incidencia acumulada de LME del 53,8% en el grupo no expuesto de las principales variables independientes, valor obtenido en un estudio previo con población similar, en el que el 93,8% (15/16) de las variables independientes presentaron una incidencia igual o superior al 53,8% en los grupos no expuestos^13^. Finalmente, se aplicó la corrección de continuidad de Fleiss al cálculo de la muestra^20^, resultando en una muestra mínima estimada de 136 recién nacidos para evaluación entre 15 y 30 días tras el alta hospitalaria.

### Variables de estudio

Se eligieron dos variables de resultado: interrupción de la LME y destete entre 15 y 30 días después del alta hospitalaria. El destete se consideró la interrupción de la LM en la semana previa al contacto con la madre o al registro en el historial médico.

Las variables de exposición se distribuyeron en bloques según su relación de proximidad con los resultados, según un enfoque teórico adaptado^13^ ^,^ ^21^. El primer bloque teórico (distal) incluía las variables de edad y educación materna, mientras que el segundo bloque (intermedio) incluía la variable experiencia previa de lactancia materna. El tercer bloque (proximal) incluía el tipo de parto (cesárea), el peso al nacer, la edad gestacional, LME al alta, el tiempo para estimular las mamas por primera vez, la edad en la que el bebé amamantó por primera vez y la duración de la hospitalización.

Se consideró censura cuando: 1) la madre no respondía a mensajes ni llamadas; 2) la madre declaró que no deseaba continuar con la investigación; 3) el bebé murió o fue trasladado a otro hospital. La madre y el bebé solo fueron excluidos del estudio en caso de retiro del consentimiento por parte de la madre.

### Instrumentos utilizados en la recopilación de datos

Parte de la recogida de datos se realizó a través de *OpenDataKit* (ODK), una plataforma que permite la finalización *offline* por *smartphones* o tabletas y el envío de los datos a un servidor cuando hay conexión a internet. Los formularios de registro de los participantes y las preguntas sobre la alimentación (tipo de leche y método de alimentación) del RN en el momento y después del alta hospitalaria fueron desarrollados por los investigadores en esta plataforma y se sometieron a una prueba piloto. En la aplicación “*AmamentaCoach*”, desarrollada por investigadores de la Facultad de Enfermería de la Universidad Estatal de Londrina (*Faculdade de Enfermagem da Universidade Estadual de Londrina*), también se incluyeron formularios sobre la alimentación del RN para facilitar la recogida de datos. Los datos se exportaron al *Power BI*.

Se preparó una agenda de investigación en una hoja de cálculo Excel para supervisar el seguimiento de los participantes. Los datos de las plataformas ODK y *Power BI* se exportaron e integraron en una hoja de cálculo *Excel*, formando una base de datos unificada.

### Procedimiento de recogida de datos

La recogida de datos consistió en tres momentos: nacimiento, alta hospitalaria y posterior alta hospitalaria. La madre elegible fue abordada entre 72 y 120 horas después del nacimiento. Tras dar su consentimiento, la invitaron a instalar la aplicación “*AmamentaCoach*” en su dispositivo móvil.

Al alta hospitalaria y entre 15 y 30 días después del alta, la madre recibió notificaciones a través de la aplicación para informar sobre el flujo y responder preguntas sobre la alimentación del lactante. Las que no respondieron por la aplicación recibieron mensajes a través de *WhatsApp* (tres intentos) y llamadas telefónicas (tres intentos). Cuando no fue posible contactarlas, se buscó la información en el historial clínico electrónico de la maternidad.

### Aspectos éticos

Se aseguraron todos los aspectos éticos previstos en la Resolución n.º 466/2012. El estudio fue aprobado por el Comité de Ética en la Investigación (n.º 27703419.8.0000.5231). 

Se invitó a las madres elegibles a participar en el estudio y, tras ser informadas sobre los objetivos y tras la aceptación, se les leyó y firmó conjuntamente el Formulario de Consentimiento Informado. En el caso de madres adolescentes, el Término de Asentimiento y el Término de Consentimiento Libre y Esclarecido fueron entregados al responsable. 

### Tratamiento y análisis de datos

Los datos fueron importados de Excel y se analizaron utilizando el *software Stata*, versión 17.0. La normalidad de las variables cuantitativas se probó mediante la prueba de Kolmogorov-Smirnov con corrección de Lilliefors. En el análisis descriptivo, las variables cuantitativas se describieron como media y desviación estándar (DE), además de la mediana, el percentil 25 (P25) y el percentil 75 (P75) debido a la falta de normalidad. Las variables cualitativas se presentaron como frecuencia absoluta (n) y relativa (%). También se realizó un análisis descriptivo de la incidencia acumulada de resultados (interrupción de la LME y destete después del alta).

A continuación, se utilizó análisis bivariado, utilizando regresión de Poisson con varianza bivariada robusta, para analizar la magnitud de la asociación entre cada variable de exposición y los resultados. Esta estrategia se utilizó para seleccionar las variables del modelo de regresión múltiple. Las variables con un valor p<0,25 en este análisis se incluyeron en modelos de regresión múltiple^22^ ^-^ ^23^. Los resultados del análisis bivariado se presentaron como Riesgo Relativo (RR) no ajustado y respectivo intervalo de confianza del 95% (IC 95%). Los resultados del modelo de regresión múltiple se presentaron como Riesgo Relativo Ajustado (RRA) y respectivo IC del 95%, considerando p< 0,05.

## Resultados

De los 291 participantes iniciales, cinco RN fallecieron, un RN fue trasladado a otro hospital, un RN no fue dado de alta del hospital, una madre no pudo descargar la aplicación para la recogida de datos y dos madres no tenían contacto en el momento del alta de la UCIN. Tras el alta hospitalaria, no hubo contacto con las madres de 42 RN, y dos madres abandonaron el estudio, sumando un total de 237 binomios madre-hijo, con datos completos de alimentación entre 15 y 30 días después del alta hospitalaria como muestra final. La Tabla 1 muestra las características sociodemográficas y clínicas de los participantes. La mayoría de las madres tenían entre 20 y 34 años (67,1%); el 67,1% estudió hasta la educación secundaria y el 82,7% vivió con su pareja. Entre las mujeres embarazadas, el 40,5% eran primíparas, el 41,4% embarazo planificado, el 76,4% tuvo seis o más visitas prenatales, el 22,4% recibió información sobre la lactancia materna (LM) y el 53,2% tenía experiencia previa con lactancia materna. En cuanto al parto, el 59,9% tuvo complicaciones, el 38,8% tuvo parto vaginal, el 40,9% tuvo contacto piel con piel dentro de las 72 horas posteriores al parto y el 41,8% amamantó en las primeras 24 horas.

**Tabla 1:** Características de los binomios madre-hijo participantes en la cohorte del estudio (n = 237). Goiânia, GO; Londrina, PR, Brasil, 2022-2023

Variables	n* (%)^†^
Edad materna (años)	
<19	28 (11,8)
20-34	159 (67,1)
>35	50(21,1)
Educación materna	
Educación primaria incompleta o completa	35 (14,8)
Educación secundaria incompleta o completa	159 (67,1)
Educación superior	43 (18,1)
Vive con la pareja	196 (82,7)
Embarazo planificado	98 (41,4)
Primíparas	96 (40,5)
Tuvo seis o más consultas prenatales	181 (76,4)
Tuvo complicaciones durante el embarazo	142 (59,9)
Recibió información sobre lactancia materna durante el embarazo	53 (22,4)
Tiene experiencia previa con la lactancia materna	126 (53,2)
Parto vaginal	92 (38,8)
Contacto piel con piel hasta 72 horas después del parto	97 (40,9)
Primer estímulo en las mamas	
<6 horas después del parto	85 (35,9)
>6 horas después del parto	121 (51,1)
no estimuló hasta el quinto día después del parto	31 (13,1)
Peso al nacer (gramos)	
> 2500	85 (35,9)
2500-1500	122 (51,5)
< 1500	30 (12,7)
Periodo em que el recién nacido amamantó por primera vez	
< 24 horas después del nacimiento	99 (41,8)
> 24 horas después del nacimiento	51 (21,5)
no amamantó hasta el quinto día de vida	87 (36,7)
Lactancia materna exclusiva al alta hospitalaria	170 (71,7)
Edad gestacional ao nacer (semanas)	
Media (Desviación estándar)	34,19 (2,2)
Mediana (Percentile 25-75)	35 (33,5-36)
Duración total de la hospitalización (días)	
Media (Desviación estándar)	14,30 (18,6)
Mediana (Percentile 25-75)	6 (4-16,5)

*n = Número; ^†^% = Frecuencia

Los RN tenían una media de 34,19 semanas de gestación y el 51,5 % pesaba entre 1500 y 2500 g. La mayoría de las madres comenzaron la estimulación mamaria seis horas después del parto (51,1 %). La hospitalización media de los recién nacidos fue de 14,3 días. En los primeros cinco días, el 32,6 % de los prematuros recibían AME, porcentaje que aumentó al 71,7 % al alta y se redujo al 64,6 % después de 15-30 días (Figura 1). La incidencia acumulada de destete precoz fue del 9,8 % (IC del 95 %: 6,5-14,2 %).

En el análisis bivariado, las variables maternas y neonatales, como la edad materna, la educación, la experiencia con la lactancia materna, el tipo de parto, el peso al nacer y la LME al alta, fueron estadísticamente significativas para la interrupción de la LME. Para el destete de la LM, las variables maternas y neonatales fueron significativas, como la experiencia con la lactancia materna, cesárea, contacto piel con piel, peso al nacer y LME al alta (Tabla 2).

**Figura 1:** Indicadores de lactancia materna exclusiva en los primeros cinco días de vida, al alta hospitalaria y 15-30 días después del alta hospitalaria de bebés prematuros (n = 237). Goiânia, GO; Londrina, PR, Brasil, 2022-2023

**Tabla 2:** Tasa de incidencia de interrupción y destete de lactancia materna exclusiva, y Riesgo Relativo Bruto (RRb) en bebés prematuros entre 15 y 30 días después del alta hospitalaria, según variables de exposición (n = 237). Goiânia, GO; Londrina, PR, Brasil, 2022-2023

Variables maternas	Interrupción de la LME*		RRb^†^ (IC95^‡^)	valor p^§||^	Destete		RRb^†^ (IC95^‡^)	valor p^§||^
	No (n=153)	Sí (n=84)			No (n=214)	Sí (n=23)		
Edad (años)								
<19	7 (25,0)	21 (75,0)	0,63 (0,32-1,23)	0,179	25 (89,3)	3 (10,7)	1,14 (0,35-3,68)	0,832
20-34	63 (39,6)	96 (60,4)	1,00		144 (90,6)	15 (9,4)	1,00	
>35	14 (28,0)	36 (72,0)	0,70 (0,44-1,15)	0,161	45 (90,0)	5 (10,0)	1,06 (0,40-2,78)	0,906
Educación								
Hasta la educación primaria	14 (40,0)	21 (60,0)	1,56 (0,81-3,00)	0,180	145 (88,7)	18 (11,3)	0,82 (0,14-4,64)	0,822
Hasta la educación secundaria	59 (37,1)	100 (62,9)	1,45 (0,84-2,51)	0,185	40 (93,0%)	3 (7,0)	1,62 (0,50-5,3)	0,420
Educación superior o más	11 (25,6)	32 (74,4)	1,00		40 (93,0)	3 (7,0)	1,00	
Vive con la pareja								
No	13 (31,7)	28 (68,3)	0,88(0,54-1,42)	0,592	36 (87,8)	5 (12,2)	1,33 (0,52-3,38)	0,552
Sí	71 (36,2)	125 (63,8)	1,14 (0,70-1,85)	0,592	178 (9,8)	18 (9,2)	1,00	
Tuvo > 6 consultas prenatales								
No	21 (37,5)	35 (62,5)	1,07 (0,73-1,60)	0,710	50 (89,3)	6 (10,7)	1,14 (0,47-2,76)	0,770
Sí	63 (34,8)	118 (65,2)	1,00		164 (90,6)	17 (9,4)	1,00	
Recibió información sobre lactancia materna durante el prenatal								
No	67 (36,4)	117 (63,6)	1,13 (0,73-1,76)	0,569	164 (89,1)	20 (10,9)	1,92 (0,59-6,22)	0,277
Sí	17 (32,1)	36 (67,9)	1,00		50 (90,3)	3 (5,7)	1,00	
Experiencia previa con lactancia materna								
No	44 (39,6)	67 (60,4)	1,25 (0,88-1,76)	0,207	96 (86,5)	15 (13,5)	2,12 (0,93-4,83)	0,071
Sí	40 (31,7)	86 (68,3)	1,00		118 (93,7)	8 (6,3)	1,00	
Parto por cesárea								
Sí	65 (44,8)	80 (55,2)	2,17 (1,40-3,37)	0,001	126 (86,9)	19 (13,1)	3,01 (1,05-8,60)	0,039
No	19 (20,7)	73 (79,3)	1,00		88 (95,7)	4 (4,3)	1,00	
Contacto piel con piel hasta 72 horas después del parto								
No	52 (37,1)	88 (62,9)	1,12 (0,79-1,61)	0,515	122 (87,1)	18 (12,9)	2,49 (0,95-6,50)	0,062
Sí	32 (33)	65 (67)	1,00		92 (94,8)	5 (5,2)	1,00	
Primer estímulo en las mamas (horas)								
<6 h después del parto	15 (17,6)	70 (82,4)	1,00		83 (97,6)	2 (2,4)	1,00	
>6 h después del parto	52 (43,0)	69 (57,0)	2,43 (1,47-4,03)	0,001	109 (90,1)	12 (9,9)	4,21 (0,96-18,40)	0,056
no estimuló hasta 5 días después del parto	17 (54,8)	14 (45,2)	3,10 (1,77-5,44)	<0,001	22 (71,0)	9 (29,0)	12,3 (2,81-54,15)	0,001
**Variables Neonatales**	Interrupción de la LME*		**RRb^†^ (IC95^‡^)**	**valor p^§||^**	**Destete**		**RRb^†^ (IC95^‡^)**	**valor p^§||^**
	**No (n=153)**	**Sí (n=84)**			**No (n=214)**	**Sí (n=23)**		
Peso al nacer (gramos)								
> 2.500	19 (22,4)	66 (77,6)	1,00		81 (95,3)	4 (4,7)	0,71 (0,22-2,31)	0,578
2.500-1.500	43 (35,2)	79 (64,8)	1,58 (0,99-2,50)	0,055	114 (93,4)	8 (6,6)	1,00	
< 1.500	22 (73,3)	8 (26,7)	3,28 (2,08-5,15)	<0,001	19 (63,3)	11 (36,7)	5,59 (2,46-12,70)	<0,001
Edad gestacional al nacer (semanas)								
<28	1 (20)	4 (80)	0,61 (0,10-3,58)	0,585	4 (80)	1 (20)	3,06 (0,49-19,03)	0,231
28-32	13 (72,2)	5 (27,8)	2,20 (1,56-3,11)	<0,001	10 (55,6)	8 (44,4)	6,79 (3,29-14,02)	<0,001
32-37	70 (32,7)	144 (67,3)	1,00		200 (93,5)	14 (6,5)	1,00	
Tiempo de hospitalización (días)								
< 7	28 (23,3)	92 (76,7)	1,00		117 (97,5)	3 (2,5)	1,00	
7-14	13 (24,5)	40 (75,5)	1,05 (0,59-1,87)	0,865	50 (94,3)	3 (5,7)	2,26 (0,47-10,9)	0,308
> 14	43 (67,2)	21 (32,8)	2,88 (1,99-4,16)	<0,001	47 (73,4)	17 (26,6)	10,62 (3,22-35,0)	<0,001
Edad a la que el recién nacido mamó por primera vez del pecho								
< 24 horas después del parto	28 (28,3)	71 (71,7)	1,00		96 (97)	3 (3)	1,00	
> 24 horas después del parto	16 (31,4)	35 (68,6)	1,11 (0,66-1,85)	0,693	46 (90,2)	5 (9,8)	3,23 (0,80-13,03)	0,099
no fue amamantado hasta el quinto día de vida	40 (46,0)	47 (54,0)	1,62 (1,10-2,40)	0,014	72 (82,8)	15 (17,2)	5,68 (1,70-19,04)	0,005
Estaba en LME* en el alta hospitalaria								
Não	55 (82,1)	12 (17,9)	4,81 (3,39-6,83)	<0,001	48 (71,6)	19 (28,4)	12,05 (4,24-34,19)	<0,001
Sim			1,00		166 (97,6)	4 (2,4)	1,00	

*LME = Lactancia materna exclusiva; ^†^RRb = Riesgo relativo no ajustado; ^‡^IC95% = Intervalo de confianza de 95%; ^§^p=0,05; ^||^Prueba del qui-cuadrado de Wald

En el análisis jerárquico múltiple (Tabla 3), la edad materna > 35 años redujo el riesgo de interrupción del lactante exclusivo en 0,30 veces. La baja educación aumentó el riesgo: 1,68 veces hasta la educación primaria y 1,79 veces hasta la educación secundaria. La estimulación mamaria tras 6 horas o ninguna estimulación aumentó el riesgo en 1,88 y 1,69 veces, respectivamente. La duración de la hospitalización aumentó el riesgo en 1,85 veces. La primera lactancia después de 6 horas aumentó el riesgo en 1,61 veces, y no haber lactancia hasta el quinto día, en 1,66 veces. No estar en LME al alta, el riesgo aumentó 3,64 veces.

En el análisis jerárquico multivariante, no estar en lactancia materna al alta aumentó el riesgo de reducción de la lactancia corporal entre 15 y 30 días después del alta en 6,13 veces, como se muestra en la Tabla 4.

**Tabla 3:** Análisis multivariante utilizando regresión de Poisson con un modelo jerárquico para la interrupción de la lactancia materna exclusiva entre 15 y 30 días después del alta hospitalaria de bebés prematuros y factores asociados (n = 237). Goiânia, GO; Londrina, PR, Brasil, 2022-2023

Variables	Modelo I		Modelo II		Modelo III	
	RRA*	IC95^†^	RRA*	IC95^†^	RRA*	IC95^†^
Bloque 1						
Edad de la madre (años)						
< 19	0,57 (0,28-1,14)		0,50 (0,25-1,02)		0,63 (0,36-1,12)	
20-34	1,00		1,00		1,00	
> 35	0,71 (0,43-1,16)		0,75 (0,45-1,25)		0,70 (0,50-1,0)^‡^	
Educación de la madre						
Hasta la educación primaria	1,75 (0,90-3,40)		1,95 (1,0-3,7)^||^		1,68 (1,00-2,80)^‡^	
Hasta la educación secundaria	1,50 (0,85-2,62)		1,6 (0,9-2,7)		1,79 (1,20-2,66)^‡^	
Educación superior o más	1,00		1,00		1,00	
**Bloque 2**						
Experiencia previa con lactancia materna						
No			1,38 (1,0-1,9)		1,26 (0,94-1,69)	
Sí			1,00		1,00	
**Bloque 3**						
Parto por cesárea						
No					1,00	
Sí					1,26 (0,85-1,88)	
Primer estímulo en las mamas						
< 6 h después del parto					1,00	
>6 h después del parto					1,88 (1,13-3,13)^‡^	
no estimuló hasta el quinto día después del parto					1,69 (0,98-2,88)^‡^	
Peso al nacer (gramos)						
> 2.500					1,00	
2.500-1.500					1,24 (0,72-2,14)	
< 1.500				0,89 (0,61-1,28)	
Edad gestacional al nacer (semanas)						
< 28					0,37 (0,10-1,32)	
28-32					0,99 (0,71-1,37)	
32-37					1,00	
Tiempo de hospitalización						
< 7 días					1,00	
7-14 días					0,97 (0,58-1,63)	
> 14 días					1,85 (1,10-3,08)^‡^	
Edad a la que mamó por primera vez del pecho						
< 24 horas después del parto					1,00	
> 24 horas después del parto					0,61 (0,38-0,99)^‡^	
no mamó del pecho hasta el quinto día de vida					0,66 (0,43-1,01)^‡|^	
Lactancia materna exclusiva al alta hospitalaria						
No					3,64 (2,45-5,40)^§^	
Sí					1,00	

*RRa = Riesgo Relativo ajustado; ^†^IC95% = Intervalo de confianza de 95%; ^‡^p<0,05 Prueba de qui-cuadrado de Wald; ^§^p<0,001

**Tabla 4:** Análisis multivariante usando regresión de Poisson con un modelo jerárquico para el destete a partir de la lactancia materna 15-30 días después del alta hospitalaria de bebés prematuros y factores asociados (n = 237). Goiânia, GO; Londrina, PR, Brasil, 2022-2023

Variables	Distal		Intermediário		Proximal	
	RRA*	IC95%^†^	RRA*	IC95^†^	RRA*	IC95^†^
Bloque 1						
Experiencia previa con lactancia materna						
No			2,13 (0,93-4,84)		1,97 (0,96-4,02)	
Sí			1,00		1,00	
**Bloque 2**						
Parto por cesárea						
No					1,00	
Sí					1,23 (0,42-3,56)	
Primer estímulo en las mamas						
< 6 horas después del parto					1,00	
>6 horas después del parto					1,75 (0,44-6,89)	
no estimuló hasta el quinto día después del parto					2,47 (0,62-9,83)	
Peso a nacer (gramos)						
> 2500					1,00	
< 1500					1,50 (0,55-4,07)	
> 2500					2,72 (0,64-11,4)	
Edad gestacional al nacer (semanas)						
< 28					0,85 (0,19-3,77)	
28-32					1,84 (0,68-4,96)	
32-37					1,00	
Tiempo de hospitalización (días)						
< 7					1,00	
7-14					1,85 (0,36-9,35)	
> 14					2,98 (0,64-13,72)	
Contacto piel con piel hasta 72 horas después del parto						
No					1,50 (0,51-4,35)	
Sí					1,00	
Lactancia materna exclusiva al alta hospitalaria						
No					6,13 (2,43-15,40)^‡^	
Sí					1,00	

*RRa = Riesgo Relativo ajustado, ^†^IC95% = Intervalo de confianza de 95%, ^‡^p<0,001 Prueba de qui-cuadrado de Wald

## Discusión

Los beneficios de la LME son diversos, tanto para la madre/familia, como para el bebé^8^ ^-^ ^10^. Para el grupo de los PT, los beneficios se extienden y pueden afectar directamente a la respuesta clínica de estos RN hospitalizados en una unidad neonatal, reduciendo infecciones, entre otras complicaciones, además de que la leche materna es esencial para el desarrollo del PT. Existen muchas dificultades para mantener a estos bebés en LM y se extienden después del alta hospitalaria^24^.

En este estudio, se observó que hubo una reducción en la incidencia de LME tras el alta hospitalaria (del 71,7% al 64,2%). Estudios en otras ciudades y países muestran diferencias en los indicadores de LME al alta y después del alta hospitalaria, lo que destaca la importancia de comprender el contexto y los factores de riesgo al comparar estos datos.

Un estudio en Goiânia-GO, con 113 PT, también mostró una disminución en la incidencia de LME 7-15 días después del alta (del 81,4% al 66,4%)^13^. En Recife-PE, un estudio transversal realizado en dos hospitales públicos con 108 PT encontró una incidencia de LME del 85,2% al alta, 75% a los 15 días y 46,3% a los 30 días^25^. Un estudio de cohorte en Río de Janeiro-RJ siguió a 1.003 bebés (el 22,5% eran PT). La prevalencia de la LME al alta hospitalaria fue solo del 14,3% entre los PT^24^.

Tras el alta hospitalaria, el índice de LME fue menor que el encontrado en el estudio transversal realizado en Recife con 108 PT^25^ y en el estudio de cohorte prospectivo realizado en Goiânia con 113 PT^13^ 15-30 días después del alta; sin embargo, fue mayor que en el estudio de Recife^25^ a los 30 días del alta hospitalaria. Todos los estudios se realizaron en hospitales *Amigo da Criança* (Amigo del Niño), y la recopilación de datos en nuestro estudio tuvo lugar durante la pandemia de COVID-19.

En Grecia, un estudio retrospectivo con 279 madres y recién nacidos, 122 (43,7%) con PT (15,4% con EG < 32 semanas y 28,3% con PT tardíos), mostró una prevalencia de LME del 58,1% en el primer mes. La duración media de la LME fue de 2 meses en los PT y de 3 meses en los a término (p=0,002). Los bebés prematuros tenían un 1,64% más de riesgo de interrumpir la LME^26^. En Shanghái (China), un estudio de cohorte con 500 PT mostró que el 19% fue amamantado un mes después del alta. Durante la hospitalización en la unidad neonatal, las madres no podían amamantar directamente y la extracción de leche humana era administrada por profesionales^27^.

En Australia, un estudio de cohorte con 270 PT tardíos mostró que el 74% de los bebés fueron dados de alta de la LME, reduciéndose al 41% a las 6 semanas de edad corregida^28^. En Suecia, un estudio con 29.445 PT (6,6% extremo, 16% muy prematuro y 18% moderado) encontró una incidencia de LME del 59% en 2004 y del 45% en 2013^29^.

Se observó que la incidencia de LME al momento del alta^28^ ^-^ ^29^ y después del alta^26^ ^-^ ^28^ fue mayor en nuestro estudio en comparación con otros países. A pesar de las diferencias, las tasas de LME disminuyen significativamente tras el alta hospitalaria, lo que pone de manifiesto los retos para mantener la LME en esta población, incluso en países que tienen una política para fomentar la lactancia materna, como Brasil y Suecia.

Estos números, aunque expresivos, siguen siendo inferiores a los recomendados por la OMS, que recomienda un 90% al alta y un 70% de LME en los primeros 6 meses^30^. Sin embargo, no existen especificaciones de estos objetivos para los PT, a pesar de la evidencia de una mayor vulnerabilidad de estos bebés al destete precoz.

Los datos globales revelaron que, entre 2015 y 2021, solo el 48 % de los lactantes menores de seis meses fueron amamantados exclusivamente en todo el mundo, y el 16 % de las muertes infantiles anuales se producen debido a una lactancia inadecuada^30^.

Una iniciativa del Fondo de las Naciones Unidas para la Infancia (UNICEF) y la OMS ha identificado prioridades para que los países protejan y promuevan la lactancia materna. Solo el 16% de los países han alineado su legislación con el Código Internacional de Sustitutos de la Leche Materna, con África y Asia a la cabeza. Solo el 9% de los países cumple la recomendación de permiso por maternidad remunerada, mientras que Oceanía no cumple con el 31%. En el 14% de los países, la mayoría de los partos ocurrieron en Hospitales Amigo del Niño; el 70% tenía programas comunitarios de lactancia materna; el 23% contaba con apoyo en emergencias; y el 43% ha recopilado datos sobre la LME en los últimos cinco años^30^. En el ranking de la Iniciativa Mundial de Tendencias en la Lactancia (*World Breastfeeding Trends Initiative)*, Brasil ocupa la posición 18, liderado por Bangladés (1º lugar)^31^.

La mejora de estos indicadores solo es posible si se identifican los factores de riesgo del destete y se planifican políticas y acciones específicas para cada contexto. En este estudio, el análisis multivariado reveló una baja educación materna como factor de riesgo y la edad materna como factor protector para la interrupción de la LME 15-30 días después del alta hospitalaria del PT. Un estudio en Maceió-AL con 161 PT con LME al alta hospitalaria encontró un resultado similar, lo que sugiere que las mujeres de este grupo de edad son más conscientes de los beneficios de la LME, a pesar de los riesgos obstétricos asociados^32^. Otro estudio con 622 mujeres puérperas en Porto Alegre-RS asoció la edad materna ≥ 35 años como factor protector para la LME. Por otro lado, las mujeres entre 26 y 34 años se enfrentan a retos relacionados con la vida laboral, como la necesidad de volver al trabajo, la brevedad del permiso de maternidad y la inseguridad financiera^33^.

Una revisión sistemática identificó 36 factores asociados con la interrupción de la LME, y se consideraron factores distales variables socioeconómicas como el lugar de residencia, la educación y la raza. La educación materna fue la variable más investigada, con baja educación frecuentemente asociada a la interrupción de la LME^21^. En nuestro estudio, la baja educación materna también se asoció con un mayor riesgo de interrupción de la LME entre 15 y 30 días después del alta hospitalaria. Sin embargo, solo investigamos dos variables distales (la educación y la edad de la madre), lo que fue una limitación del estudio.

Una baja educación puede dificultar la comprensión de las directrices y el mantenimiento de la LME^34^. Además, las madres con más años de educación posiblemente tienen mayor control en el entorno laboral, recibiendo más apoyo y ánimo para la LME exclusivamente durante más tiempo^35^.

Las acciones educativas para mejorar la comprensión de la lactancia materna son efectivas para el inicio y el mantenimiento de la LME, especialmente si se llevan a cabo durante el embarazo en unidades básicas de salud o clínicas ambulatorias de alto riesgo. Dos estudios mostraron un aumento en el conocimiento tras acciones educativas (exposiciones dialogadas, vídeos, carteles), pero no analizaron la mejora en las tasas de LME^36^ ^-^ ^37^.

El análisis multivariante reveló asociaciones con varias variables proximales: tiempo de estimulación mamaria por primera vez, edad de la primera lactancia, duración de la hospitalización y LME al momento del alta. La ausencia de LME al momento del alta fue la variable con mayor riesgo de interrupción de la LME (3,64 veces mayor) y de destete (6,13 veces mayor) tras el alta.

Un estudio de cohorte con 12 países (Albania, Brasil, Bulgaria, Chipre, Chile, Grecia, Israel, Malta, Portugal, España, Turquía y Reino Unido) investigó el impacto de la pandemia en los factores que interfieren con las tasas de lactancia materna, analizando datos de 5.612 mujeres de países de ingresos altos y medios altos. Los factores de riesgo para la LME incluyeron la primiparidad, la edad del bebé, la prematuridad, el ingreso en la Unidad de Cuidados Intensivos Neonatales (UCIN), la falta de apoyo a la lactancia materna durante el periodo prenatal y tras el alta, y el tratamiento psiquiátrico^38^.

Otros estudios también han encontrado una asociación entre la interrupción de la LME en PT y la duración de la hospitalización^24^ ^,^ ^39^. En nuestro estudio, la mayoría de los bebés estuvieron hospitalizados menos de 15 días. Los estudios longitudinales con un tiempo de seguimiento más largo pueden aclarar mejor esta relación.

Una política nacional eficaz y de bajo coste, que contribuye a la reducción del tiempo de hospitalización es el Método Canguro (MC), que es una estrategia prioritaria para bebés PT y de bajo peso. Según el Ministerio de Salud, uno de los criterios para el alta hospitalaria en la segunda fase del MC es la LME. El MC reduce el tiempo de separación entre el RN y los padres, facilita el vínculo familiar, ayuda a la estabilización clínica del RN y asegura la estimulación y el mantenimiento de la lactancia materna. Tras el alta, estos bebés reciben un seguimiento especializado por parte del equipo de una Unidad de Salud Básica, en colaboración con el hospital maternidad de origen, durante las primeras semanas en casa^40^.

Hay evidencia de que la lactancia materna temprana y el contacto piel con piel en PT^26^ y a término^41^ contribuyen al mantenimiento de la LME. En este estudio, menos de la mitad de los PT realizaron el MC en un plazo de 72 horas tras el parto. Cabe destacar que solo una UCIN participante tiene un protocolo basado en la evidencia con criterios para el inicio de la posición canguro, generalmente subjetivos, vinculados a la estabilidad clínica del bebé, lo que puede retrasar el inicio de este contacto.

Algunos estudios han asociado el tipo de alimentación del PT en la UCIN y la producción de leche materna con la duración de la LME^26^ ^-^ ^27^ ^,^ ^41^, pero no encontramos estudios sobre la edad a la que el PT fue amamantado por primera vez asociados con la interrupción de la LME. Evidencias científicas indican que la estabilidad del bebé debe ser el único criterio para iniciar la lactancia, ya que los PT son competentes para amamantar temprano^42^. En nuestro estudio, el 87 (36,7%) de los PT no amamantaron hasta el quinto día de vida.

La experiencia previa con la lactancia materna fue el único factor intermedio en el análisis multivariante jerárquico. La mitad de las mujeres posparto (53,2%) ya tenía experiencia previa con la lactancia materna. Esta variable mostró una asociación con la interrupción de la LME en el análisis bivariado, pero no fue significativa en el análisis multivariante, a diferencia de otros estudios^26^ ^,^ ^41^.

Una revisión sistemática de 15 estudios con 16.579 madres encontró que la experiencia previa de lactancia materna está asociada con el inicio y la duración de la lactancia materna posterior. La calidad de esta experiencia, aunque satisfactoria y prolongada, influyó positivamente en la duración de la lactancia materna^43^. Futuros estudios deberían investigar la calidad y duración de la experiencia previa de lactancia materna como factores de riesgo para la continuidad de la LME.

La mayoría de las madres realizaron la primera estimulación en los pechos 6 horas después del parto (51,1%) o no lo hicieron hasta la entrevista (13,1%). Un estudio con 263 madres de PT encontró un mayor riesgo de ausencia de LME al alta, cuando el ordeño comenzó tras 48 horas^39^. Un estudio investigó los factores asociados con el inicio tardío de la extracción de leche materna (>6 horas después del parto) en 129 madres de PT. Se identificaron como factores de riesgo para los ordeños tardíos la experiencia previa en la UCIN, el sexo masculino del bebé, el bienestar psicológico materno comprometido, la cesárea y la edad gestacional al nacer^44^.

Se recomienda que las orientaciones y los cuidados adecuados para las mujeres puérperas para la extracción de leche se realicen durante el embarazo y en las primeras 3-6 horas tras el parto, o lo antes posible^7^ ^,^ ^45^. La madre debe recibir instrucciones y demostraciones sobre cómo extraer leche manualmente o mediante extractor^42^. La alimentación con leche materna impacta significativamente en la colonización intestinal temprana del PT, protegiendo contra la enterocolitis necrotizante^45^.

La planificación y articulación de acciones entre los sectores hospitalarios son esenciales para asegurar el inicio temprano de la extracción de ordeño y facilitar el suministro eficiente de leche materna al PT. En cuanto al destete de la LM 15-30 días después del alta hospitalaria del PT, el análisis multivariante jerárquico identificó solo una variable de riesgo significativa: no estar en LME al momento del alta hospitalaria (riesgo 6,13 veces mayor). Estudios previos corroboran que la ausencia de LME al momento del alta está asociada con un mayor riesgo de destete precoz^25^ ^,^ ^46^. La oferta de leche materna en la primera alimentación enteral del PT, cuando se realiza de forma complementaria durante la hospitalización, se asoció con el destete tras el alta hospitalaria en otros estudios, ya que, cuando se prescribió durante la hospitalización, las mujeres puérperas cuestionaron su capacidad para amamantar^24^ ^,^ ^26^ ^-^ ^27^. 

Varios obstáculos tras el alta hospitalaria pueden dificultar el mantenimiento de la LME, como la adaptación a la rutina doméstica, la percepción materna de baja leche, la falta de apoyo familiar y profesional, el regreso temprano al trabajo, la introducción temprana de alimentos complementarios y la falta de información sobre la importancia de la LME^24^ ^-^ ^25^ ^,^ ^41^. Los estudios futuros que sigan a estos bebés prematuros durante más tiempo tras el alta son importantes para investigar otros factores que interfieren con el destete temprano.

Los profesionales de salud que trabajan en unidades neonatales deberían implementar intervenciones individualizadas para promover el desarrollo del PT, protegiendo y fomentando la lactancia materna desde el principio, facilitando el contacto piel con piel temprano y promoviendo su continuidad^47^.

Entendemos que las diferencias culturales y las políticas de salud influyen en los resultados de la lactancia materna de bebés prematuros, integrando las evidencias internacionales y experiencias locales, como el Método Canguro en Brasil y la variabilidad en la práctica del contacto piel con piel. Estas disparidades revelan un escenario complejo de desigualdad en las tasas de LME. En vista de esto, los autores defienden la urgencia de la investigación longitudinal y prospectiva en enfermería, orientada a evaluar el impacto de factores culturales y sistémicos y las prácticas de cuidado en la duración de la LME tras el alta. También destacan la importancia de investigar cómo la experiencia previa de lactancia materna y el contexto de cuidados afectan a las decisiones maternas, además de probar intervenciones de enfermería basadas en la ciencia y adaptadas culturalmente.

Los resultados de este estudio refuerzan que la implementación de acciones basadas en la evidencia científica y las políticas existentes puede mejorar los indicadores de LME y reducir el destete precoz tras el alta hospitalaria del PT, favoreciendo la LME hasta los seis meses de edad.

Se destaca el papel crucial de la enfermera en la dirección de la atención integral del PT y de su familia, tanto en la UCIN como tras el alta hospitalaria. La planificación y ejecución de acciones para promover la lactancia materna son responsabilidades fundamentales de estos profesionales^47^.

La atención proporcionada a los PT en hospitales con el título de Amigo del Niño y la implementación del MC probablemente contribuyeron a los buenos resultados de la lactancia materna al alta. Sin embargo, la integración con la Atención Primaria de Salud es crucial para mantener las directrices y acciones de promoción y protección de la LME en casa, reduciendo así su tasa de destete.

El contexto social de la mujer puerperal, aunque no se investiga en este estudio, también es determinante. La red de apoyo social puede facilitar la continuidad de la lactancia materna a medida que el bebé crece y surgen nuevos desafíos, como volver al trabajo. Las políticas y legislación que protejan la lactancia materna son esenciales para crear un entorno favorable y acogedor, promoviendo no solo la salud de las mujeres puérperas y lactantes, sino también la igualdad en la sociedad.

Este estudio presenta algunas limitaciones que deben tenerse en cuenta a la hora de interpretar los resultados. El muestreo no probabilístico por conveniencia pudo haber introducido sesgo de selección. Además, el criterio de inclusión que requería acceso a *smartphones* con sistema *Android* para monitorización podría haber potenciado estos sesgos. Aunque estas estrategias fueron necesarias para superar las dificultades prácticas de acceder a una población aleatoria de recién nacidos prematuros y para ampliar las posibilidades de permitir el contacto con los participantes durante el seguimiento (a través de la aplicación y *WhatsApp*), pueden limitar la generalización de los resultados a otros contextos y poblaciones de RN. Además, la recopilación de datos solo se realizó en dos regiones del país e incluyó solo dos hospitales públicos y con el título de Amigo del Niño. Dado el componente cultural relacionado con la lactancia materna, es importante considerar también estos factores, que podrían reducir aún más la generalización del estudio. Otra limitación relevante se refiere al hecho de que la recopilación de datos se realizó solo en dos regiones del país, incluyendo exclusivamente dos hospitales públicos certificados como Amigos del Niño. Teniendo en cuenta la influencia de factores culturales en las prácticas de lactancia materna, esta característica del estudio también puede restringir la generalización de los hallazgos. Sin embargo, es importante destacar que la selección de hospitales con esta certificación representa un perfil especialmente relevante para la formulación de políticas públicas nacionales en el ámbito de la lactancia materna.

En cuanto al sesgo de supervivencia, tuvimos una pérdida total del 18% en el seguimiento, siendo la dificultad de contacto la causa principal de las pérdidas (n=42). Es posible que estas mujeres puérperas tuvieran mayores dificultades para amamantar y, por tanto, no quisieran compartir su fracaso en la discontinuidad de amamantar. 

## Conclusión

La mayoría de los PT estaban en LME al alta hospitalaria, con una disminución del 35,3% entre 15 y 30 días después del alta. Los factores asociados a la suspensión de la LME 15-30 días después del alta hospitalaria incluyeron la edad materna >35 años; educación materna (hasta educación primaria; hasta educación secundaria); primera estimulación de los pechos tras el parto (>6 h; no estimulada); duración de la estancia hospitalaria >14 días; edad a la que amamantó por primera vez (>24 h después del parto; no amamantó); y LME al alta hospitalaria. En el destete, solo la variable de no estuviera en lactancia materna al alta hospitalaria mostró asociación.

Los resultados indicaron que los indicadores de LME en lactantes prematuros eran comparables o superiores a los encontrados en estudios nacionales e internacionales, pero estaban por debajo de los objetivos establecidos por organizaciones internacionales para la prevención de muertes infantiles y neonatales.

Los factores de riesgo identificados para la interrupción de la LME y el destete del lactante destacan variables que pueden modificarse con una atención de calidad basándose en la evidencia científica, que implica acciones articuladas entre profesionales y sectores que atienden a mujeres embarazadas y madres de PT durante el proceso de lactancia materna.

La intervención temprana, el apoyo familiar, un entorno hospitalario favorable y políticas de apoyo son esenciales para promover y mantener la lactancia materna en PT tras el alta, asegurando una atención adaptada a las necesidades de esta población vulnerable.

## Declaración de Disponibilidad de Datos:

Los conjuntos de datos relacionados con este artículo estarán disponibles previa solicitud al autor correspondiente.
